# Excess mortality in Mainland China after the end of the Zero COVID policy: A systematic review

**DOI:** 10.1017/S0950268826101022

**Published:** 2026-02-06

**Authors:** Isaac Chun-Hai Fung, Hai Liang, Kelli J. Pierce, Alicia N. M. Kraay, Kin On Kwok, Andrei R. Akhmetzhanov, Frank E. Baiden, H. Juliette T. Unwin, Francis B. Kengne, Faharudeen Alhassan, Gerardo Chowell

**Affiliations:** 1 Department of Biostatistics, Epidemiology and Environmental Health Sciences, Jiann-Ping Hsu College of Public Health, Georgia Southern University, USA; 2 School of Journalism and Communication, The Chinese University of Hong Kong, Hong Kong; 3 Gates Foundation, USA; 4 The Jockey Club School of Public Health and Primary Care, The Chinese University of Hong Kong, Hong Kong; 5 Hong Kong Institute of Asia-Pacific Studies, The Chinese University of Hong Kong, Hong Kong; 6 Department of Infectious Disease Epidemiology, Imperial College London, UK; 7 Institute of Epidemiology and Preventive Medicine, National Taiwan University College of Public Health, Taiwan; 8 Office of the Dean, University of Health and Allied Sciences School of Public Health, Ghana; 9 School of Mathematics, The University of Bristol, UK; 10 Department of Population Health Sciences, School of Public Health, Georgia State University, USA; 11 Department of Applied Mathematics, College of Applied Sciences, Kyung Hee University, Korea

**Keywords:** China, COVID-19, mortality, SARS-CoV-2, vital statistics

## Abstract

After the Zero COVID policy ended on December 7, 2022, ~90% of mainland Chinese were infected in a COVID-19 wave. This systematic review synthesized research estimating excess mortality during that wave in mainland China. We searched seven databases in May 2024 and updated our search in July–August 2025. Peer-reviewed research (Chinese or English), published since January 1, 2023, estimating excess deaths in the COVID-19 wave post-Zero-COVID was included. Risk of bias was assessed using a modified Newcastle-Ottawa Scale. Two authors independently conducted abstract screening, full-text review, data extraction, and risk-of-bias assessment. Seven articles were included. Two studies analysed the death records of a town and a district in Shanghai, estimating the excess mortality rates of 153.6% and 174.3%, respectively. Using indirect methods, four studies estimated national excess mortality (range: 0.71–1.87 million). Another study estimated excess mortality in Taiyuan. Studies used diverse methods to estimate excess deaths, resulting in widely varying and uncertain estimates. Choice of reference period, seasonality, and other factors affect expected mortality estimates.

## Key results

- A systematic review was conducted on the literature on excess mortality in mainland China during the COVID-19 wave after the Zero COVID policy ended.
- Two studies analysed the death records of a town and a district in Shanghai estimating the excess mortality rates of 153.6% and 174.3% respectively.
- Using indirect methods four studies estimated national excess mortality (range: 0.71–1.87 million).
- Diverse methods applied to estimate excess deaths in this setting and consequent different and uncertain results were identified.

## Introduction

A stringent public health and social measures protocol, primarily consisting of nonpharmaceutical interventions, was imposed in mainland China from 23 January 2020 to 6 December 2022. This protocol, known as the Zero COVID policy, mandated obligatory facemask wearing, social distancing, contact tracing, isolation of patients, and quarantine of exposed individuals and inbound travellers. In addition, mass testing and regional lockdowns were imposed in locations with recent clusters of cases [1, 2]. While implementing the Zero COVID policy, the government rolled out locally developed inactivated vaccines and one billion people were fully vaccinated by September 2021 [3]. Two doses of CoronaVac (Sinovac) had an estimated real-world effectiveness of 55% against mortality among the ≥80-year-olds before the emergence of the Omicron variant [4]. However, the inactivated vaccine was found less effective against the Omicron variant [5]. The government did not authorize foreign-made mRNA vaccines for emergency use in mainland China following the emergence of the Omicron variants, except for expatriates residing there.

Anecdotal evidence suggested that the Omicron variant was spreading nationwide, leading to large-scale lockdowns and mass testing in many locations [1, 6]. By November 2022, the government was reconsidering the Zero COVID policy [1, 6] and abandoned it on 7 December 2022 [7], when all the public health and social measures prescribed by the Zero COVID policy were lifted [6]. People could now resume their daily routines and conduct business as in the pre-pandemic era. Subsequently, there was a huge COVID-19 wave in mainland China. According to Du et al., the Chinese Center for Disease Control and Prevention (China CDC) sentinel surveillance suggested that from 16 December 2022 to 19 January 2023, approximately 9 in 10 people in mainland China were infected with COVID-19 [8, 9]. However, only ~81,000 in-hospital deaths were officially confirmed by China CDC from 9 December 2022 through 30 January 2023 [10, 11]. The COVID-19 wave subsided by February 2023.

Excess mortality is a term used in epidemiology and public health that refers to the number of deaths from all causes during a crisis above and beyond what we would have expected to see under ‘normal’ conditions [12]. The estimation of excess mortality requires the enumeration or estimation of both the true number of deaths during the event and the expected number of deaths should the event not happen. Their difference is the excess mortality [13].

This systematic review aims to synthesize peer-reviewed research articles that estimated excess mortality in mainland China during the COVID-19 wave after the end of the Zero COVID policy on 7 December 2022. In our Supplementary Materials, we also review research articles that estimated mortality but not excess mortality.

## Methods

Two coauthors systematically searched for peer-reviewed research articles (in Chinese or English) in seven bibliographic databases: PubMed and Web of Science Core Collection (WoS) on 9 May 2024, and CNKI (China National Knowledge Infrastructure), Wanfang, Sinomed, Chinese Medical Journal Full Text database, and Chaoxing, on 16 May 2024. The keyword combinations included ‘COVID-19 OR SARS-CoV-2’ AND ‘China’ AND ‘(Excess Death*) OR (Excess Mortality)’; the equivalent Chinese search terms are provided in Supplementary Text S1. We limited our search to papers published on or after January 1, 2023. After deduplication, two coauthors applied our inclusion and exclusion criteria (Supplementary Table S1) to screen the titles and abstracts of the retrieved items and reviewed full texts. Papers excluded after full-text review are listed in Supplementary Table S2. Only research articles, written in English or Chinese, that included estimation of excess deaths in mainland China due to the end of the Zero COVID policy at the population level, were included. Data were extracted from the included studies. The risk of bias in the included studies was assessed using a modified Newcastle-Ottawa Scale (NOS) that we adapted from Mata et al. [14] (Supplementary Table S3). Studies with 6+ (out of 10) points were deemed to have a low risk of bias. Title/abstract screening, full-text review, data extraction, and risk of bias assessment were performed independently by two co-authors (Chinese papers: first and co-first authors; English papers: first and second authors). See Supplementary Text S1 for further details. A PRISMA 2020 Checklist [15] is provided as part of the Supplementary Materials. The search was updated on 27 July 2025 (PubMed and WoS) and from 31 July to 3 August 2025 (the 5 Chinese databases). This update yielded no additional studies meeting inclusion criteria.

## Results

**Figure 1.**
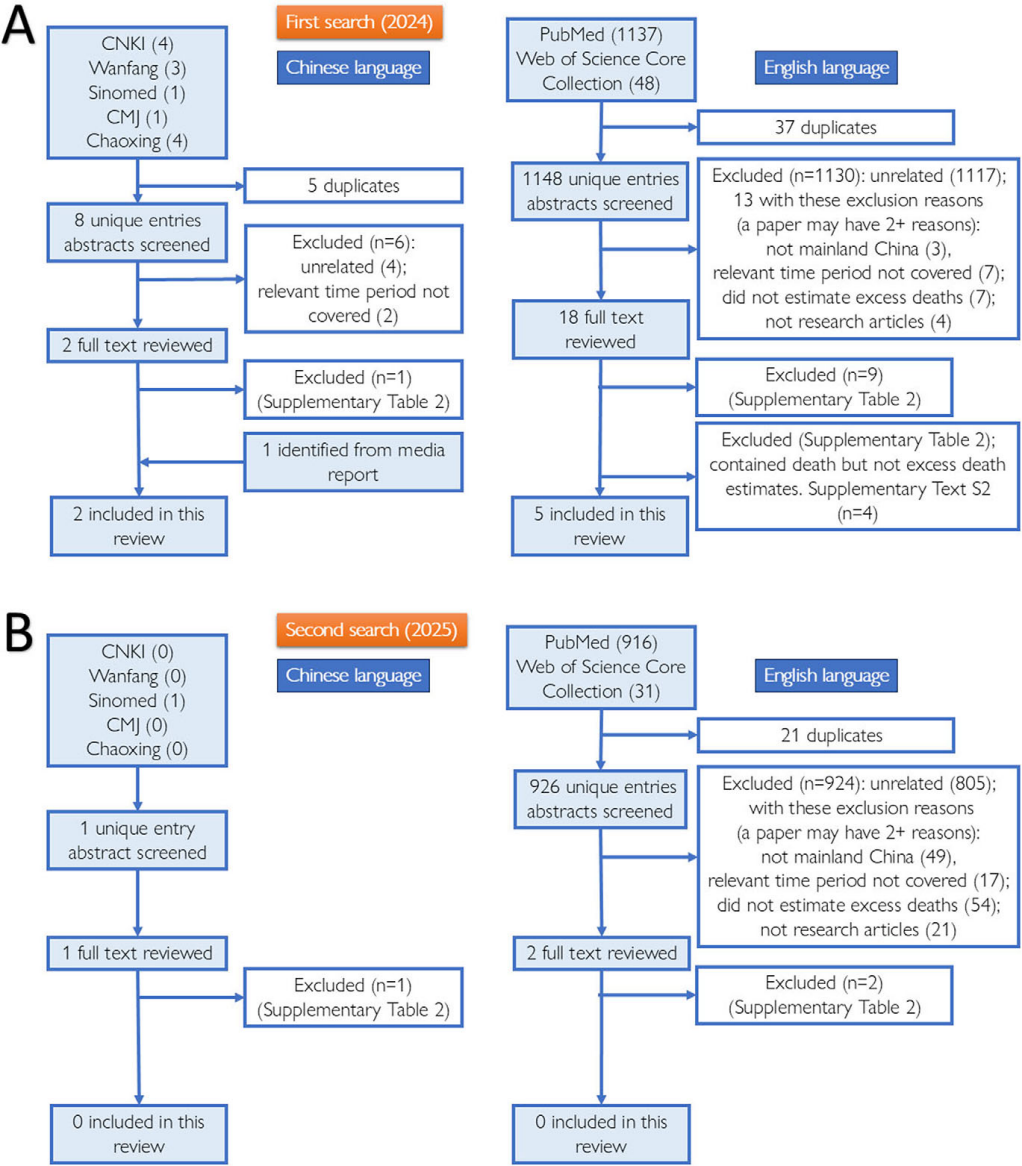
Schematic presenting the steps taken to identify literature for this systematic review: the database search, deduplication, abstract screening, full-text review, and inclusion and exclusion of literature. Panels A and B present the process and results of our first (2024) and second (2025) search, respectively. Search updated July–August 2025; no additional inclusions. PRISMA counts reconcile with Supplementary Text S1.

Seven articles [11, 16–21] were included in this systematic review (Figure 1, Tables 1, 2). Six additional articles that did not meet our inclusion criteria but provided information about estimated or projected death counts were reviewed in Supplementary Text S2–S3 [9, 22–26]. Two Chinese articles estimated excess mortality in unnamed subdivisions of Shanghai using official death records [20, 21], and five English articles estimated excess mortality in all or some populations in mainland China using other datasets [11, 16–19]. Tables 1, 2 summarize the studies and their excess mortality estimates. Below is a summary of each paper, with a detailed discussion of each paper’s methods and results, as given in Supplementary Text S1.

**Table 1.** Data sources of the seven included articles, their reference period (with which the expected number of deaths was derived), and the time period in which excess deaths were calculated

Paper	Reference period	Time period for excess deaths	Data sources	Modelling of reference period	Seasonality considered	Age considered (years)	Sex considered	Language
Zhang [21]	Dec 2015–Nov 2022	Dec 2022–Jan 2023	Official death records	SPSS 22.0 Time Series Modeler’s Method: Expert Modeler (default setting)	Yes	Age-stratified excess death estimates: <60, 60–69, 70–79, 80–89, ≥90	Sex-stratified estimates	Chinese
Zhang [20]	16 Dec 2019–6 Dec 2022	7 Dec 2022–23 Feb 2023	Official death records	SPSS 22.0 Time Series Modeler’s Method: Expert Modeler (default setting)	Yes	Age-stratified excess death estimates: ≤44, 45–59, 60–74, 75–89, ≥90	Sex-stratified estimates	Chinese
Jha [17]	2012–2019 (average monthly deaths expected); Jan 2020–Nov 2022 (family deaths among survey participants to calculate death rates)	Dec 2022–Jan 2023	Self-reported data from a survey of a nationally representative sample of participants	National annual age-specific death rates (2012–2019), divided by 12 to obtain the average monthly rates.	No	Excess death estimate for ≥60	Not for excess death estimates. Sex variable in the regression model.	English
Huang [16]	2018–2019	Dec 2022–Feb 2023	Baidu index, validated against all-cause mortality	Monthly-mean adjusted Baidu index time series to estimate the daily expected case count (same value over 3 months). The method was validated using 2011–2017 data.^a^	No	No age stratification	No	English
Raphson and Lipsitch [11]	Every Dec–Jan, Dec 2017–Jan 2022	Dec 2022–Jan 2023	Obituary data of senior (≥80-year-old) members of the Chinese Academy of Engineering	Mean deaths divided by mean number alive at the beginning of December and January in the reference period	Yes	Age-stratified: <80 and ≥80 in calculation process	Yes, sex-stratified in the calculation process	English
Xiao [19]	Jan 2016 –Dec 2019	Dec 2022–Jan 2023	Baidu index; obituary data of three universities. Validation using Beijing and Heilongjiang-specific data before extrapolation to the rest of mainland China	Interrupted time series design, segmented negative binomial regression model	No	Excess death estimate for ≥30	No	English
Wang [18]	2017–2019, and 2021 (2020 excluded)	2022	Official government sources for demographics, regional area, and reported case count. Did not provide the source for the actual death count data	Linear model between death count and population size; assumed population growth follows a logistic population growth model	No	No age-stratification	No	English

a Huang et al. [16] applied a regression model of natural log-transformed annual mortality data and natural log-transformed Baidu index data, plus a quadratic time trend, to 2011–2017 data to confirm the association between the two metrics. The national monthly-mean adjusted Baidu index time series was a population-weighted average of each province’s index.

**Table 2.** Location (population) of the excess death estimates, reported/estimated number of deaths, expected number of deaths, and excess deaths reported in the 7 included articles

Paper	Location and population of the excess death estimates	Reported or estimated number of deaths	Expected number of deaths	Excess death (excess mortality rate)^a^	Subnational excess mortality estimates provided
Zhang [21]	All residents in a town (*zhèn*) in Shanghai	317 (reported)	125	192 (153.60%)	A town in Shanghai only
Zhang [20]	All residents in a district (*qū*) in Shanghai	20,990 (reported)	7651.39	13,338.61 (174.33%)	A district in Shanghai only
Jha [17]	All residents in the whole mainland China	2.7 million (estimated)	1.35 million	1.35 million (100%)	No
Huang [16]	All residents in the whole mainland China	2,909,805^b^ (estimated)	2,196,900^b^	712,905 (32.45%)	For each of the 31 administrative subdivisions of mainland China
Raphson and Lipsitch [11]	All urban dwellers in the whole mainland China	n/a	n/a	917,000 [95%CI, 425,000 to 1.45 million]	For urban areas of Mainland China only
Xiao [19]	All ≥30-year-olds in the whole mainland China	3,172,102 (estimated)	1,300,409	1,871,693 [95%CI, 714,580 to 4,432,957] (143.93%) or 1.33 per 1000 population	For each of the 31 administrative subdivisions of mainland China
Wang [18]	All residents in Taiyuan City, Shanxi	33,863^c^	28,976–32,976	887 (2.69%) to 4887 (16.87%)	Taiyuan City only

CI, confidence intervals.

*Notes*: There are 31 administrative subdivisions in mainland China, including 22 provinces (*shěng*), 5 ‘autonomous regions’ (*zìzhìqū*) and 4 direct-administered municipalities. Shanghai is a direct-administered municipality (*zhíxiáshì*), which is equivalent to a province. A district (*qū*), equivalent to a county, is a subdivision of the municipality. Within a district, there are township-level divisions, i.e., subdistricts (*jiēdào*) and towns (*zhèn*).

a Excess mortality rate is calculated by dividing excess death numbers by expected death numbers and multiplying by 100%.

b The expected death per day = 24410 (personal communication, Prof. Oliver Zhen Li). Given a 90-day period (Dec 2022–Feb 2023), we calculated the expected number of deaths as 2,196,900. Given the reported excess death count of 712,905, we calculated the estimated number of deaths (2,909,805) and the excess mortality rate (712905/2196900=32.45%). Neither of these was directly reported in Huang et al. [16].

c Wang et al. (2024) did not explain how they estimated or obtained the reported number of deaths in Taiyuan, but they presented it in their paper as if it was a reported number from Taiyuan Municipal People’s Government [18].

Zhang et al. (2023) reported that in an unnamed town of 66,703 people, 317 deaths were registered in 2 months (December 2022 and January 2023). The authors estimated 125 (95% CI, 103–148) expected deaths (reference period: December 2017–November 2022), and therefore 192 (95% CI, 169–214) excess deaths. This gave an excess mortality rate of 153.60% (192/125) [21]. Zhang et al. (2024) reported that in an unnamed district of Shanghai, 20,990 deaths were registered from 7 December 2022 through 23 February 2023. The authors estimated the expected and excess deaths as 7651.39 and 13,338.61, respectively (reference period: 16 December 2019–6 December 2022). This gave an excess mortality rate of 174.33% (13,338.61/7651.39) [20].

Jha et al. estimated that there were 2.7 million deaths in mainland China, of which 1.35 million were excess deaths (excess mortality rate of 100%) between December 2022 and January 2023 (reference period: 2012–2019; and January 2020–November 2022) [17]. Raphson and Lipsitch estimated 917,000 (95% CI, 425,000–1.45 million) excess deaths among urban dwellers of both sexes in mainland China between December 2022 and January 2023 (reference period: 5 consecutive December–January periods, December 2017–January 2022) [11]. Huang et al. estimated 2,909,805 deaths in mainland China between December 2022 and February 2023. With the expected deaths of 2,196,900 (reference period: 2018–2019), they estimated 712,905 excess deaths (excess mortality rate of 32.45%) [16]. Finally, Xiao et al. estimated that among ≥30-year-olds in mainland China, there were 3,172,102 deaths between December 2022 and January 2023. The expected death count was estimated to be 1,300,409 (reference period: 2016–2019) and so the excess death count was 1,871,693 (95% CI, 714,580–4,432,957) with an excess mortality rate of 143.93% [19].

Wang et al. reported 33,863 deaths in Taiyuan city in 2022. With the expected death count between 28,976 and 32,976 (reference period: 2017–2019 and 2021), the excess death counts in Taiyuan were estimated to be between 887 and 4887. Excess mortality rates were between 2.69% (887/32,976) and 16.87% (4887/28,976) [18].

### Methods for estimating excess mortality

SPSS 22.0 Time Series Modeler’s Method: Expert Modeler (default setting) was applied by Zhang et al. to administrative death counts in both their studies to estimate the expected death counts [20, 21]. The five English articles used different datasets and methods to estimate excess mortality without access to the official death records. Jha et al. conducted a survey from a nationally representative sample and asked for self-reported family members’ deaths to estimate death rates over time since the beginning of the pandemic [17]. Huang et al. [16], Raphson and Lipsitch [11], and Xiao et al. [19] used indirect methods to estimate the relative increase in mortality compared with baseline (however it was defined), using either internet search volumes (Baidu index) of mortality-related keywords and/or obituary count from academic institutions. Wang et al. estimated Taiyuan City’s expected death count in 2022 using linear regression of the city-wide death rate in 2017–2019 and 2021 [18] (Tables 1, 2).

### Risk of bias assessment

We performed a risk of bias assessment using a modified NOS (Supplementary Text S1). The two Chinese papers scored 10 out of 10 points. The other five papers scored <6 points, indicating a high risk of bias (Supplementary Table S4). NOS scores are reported descriptively and complemented by a qualitative appraisal tailored to modelling studies. No study was excluded based on its NOS score.

### Studies estimating COVID-19 death counts only

In Supplementary Text S2, we reviewed four excluded articles that estimated the COVID-19 death count but not the excess death count of the COVID-19 wave after the end of the Zero COVID policy. Amemiya et al. used age-stratified final size equations to estimate 1.44 million COVID-19 deaths in mainland China [22]. Cheng et al. estimated a COVID-19 death rate of 83.7 per 100,000 persons (about 1.18 million deaths) in mainland China using an individual-based transmission model [23]. Du et al. estimated 1.41 million (95% CrI, 1.14–1.73 million) COVID-19 deaths in mainland China again using an individual-based model [9]. Ioannidis et al. estimated the age-specific infection-fatality ratios (IFRs) in Hong Kong and South Korea and then applied them to mainland China. In their main scenario, their projected COVID-19 death count was 249 thousand using the Hong Kong IFR or 153 thousand using the South Korea IFR [24].

In Supplementary Text S3, we summarized two 2022 studies that did not meet our inclusion criteria, but they were often cited by papers reviewed here. In May 2022, Cai et al. published their age-structured stochastic compartmental model exploring different intervention scenarios with mortality prediction and recommended how mainland China could open up safely in a scheduled manner. Their baseline scenario projected 1.55 million COVID-19 deaths (1.10 per 1000) in mainland China over 6 months [25]. Meanwhile, on 14 December 2022, Leung et al. released a medRxiv preprint. Their age-structured meta-population compartmental model explored how this pandemic wave would unfold in real time under several scenarios. Under Scenario 1 (status quo), 568 to 770 per million COVID-19 deaths in mainland China were projected [26]. Applying this rate to the same age structure as in Cai et al. [25], this would mean a cumulative COVID-19 death count of 0.80 million to 1.09 million.

Of note, studies in Supplementary Texts S2 and S3 are summarized qualitatively and were not included in the excess-mortality synthesis to preserve its pre-specified scope.

### Official reports of in-hospital COVID-19 deaths

Approximately 81,000 in-hospital COVID-19 deaths were reported by China CDC from 9 December 2022 through 30 January 2023 (extracted by Raphson and Lipsitch [11] from the original source [10]).

## Discussion

This systematic review covers seven peer-reviewed articles that estimated excess deaths in mainland China during the COVID-19 wave following the end of the Zero COVID policy on 7 December 2022. We identified diverse methods applied by these studies and their consequent results, which varied greatly and with great uncertainty.

Two Chinese studies analysed Shanghai’s official death registers, identifying 317 deaths in 2 months in an unnamed town of 66,703 (excess mortality rate=192/125=153.60%) and 20,990 deaths in 2.5 months in an unnamed district (excess mortality rate = 13,338.61/7651.39 = 174.33%), respectively [20, 21]. Four of the five included English papers provided national estimates of excess deaths [11, 16, 17, 19], ranging from ~713 thousand (all ages) [16] to 1.87 million (≥30 years of age) [19]. Additional studies estimating mortality but not excess mortality are summarized in Supplementary Texts S2 and S3.

### Assessment of methods

Excess death estimates are the difference between the actual and expected death counts; a lower expected death count gives a higher excess death estimate. For example, Xiao et al. estimated 1.87 million excess deaths (3.17 million–1.30 million) [19]; Jha et al. estimated 1.35 million excess deaths (2.70 million–1.35 million) [17]. In contrast, Huang et al. estimated only 0.7 million excess deaths (2.9 million–2.2 million) because the expected death count was as high as 2.2 million [16].

It is important to note that the actual all-cause mortality estimates of Jha et al. [17] (2.7 million), Huang et al. [16] (2.9 million), and Xiao et al. [19] (3.2 million) were higher than the estimates (or projections) of studies that modelled infection counts and estimated COVID-19 deaths based on IFRs (Supplementary Texts S2 and S3). The latter ranged from 249 thousand in Ioannidis et al. [24] to 1.55 million in Cai et al. [25].

#### Methodology of expected mortality estimation

In the field of methodology of excess mortality estimation, recent discussion focuses on estimating *expected* death counts, given that reported mortality data are used in those excess mortality studies. For example, Nepomuceno et al. highlighted that the choice of mortality indices (and their age-standardization), the methods used to estimate the baseline, the reference period, and the data time unit (weekly versus monthly) all affected the expected death count [27]. Levitt et al. noted the impact of the reference period, the projected period with which excess deaths were estimated, and the time patterns and their forms (e.g., if overall mortality increases or decreases over time and if it is linear or spline fit) on excess death estimates [28]. Levitt et al. also highlighted the importance of age structure and the analytic decision of whether changes in age structure should be accounted for, because mortality rates increase as populations age over time despite improved healthcare, elevating the expected death counts [29]. Ioannidis et al. [30] reviewed nine key issues in excess death estimation that have the potential for improvement, namely, adjusting for changing population structure, adjusting for changes in other high-risk indicators, completeness corrections, sensitivity to modelling choice (including choice of reference period and statistical models), post hoc corrections not based on pre-specified rules, transparency of model and model performance, underestimation of uncertainty, excess death estimates per risk strata, and causal misinterpretation.

Among the studies reviewed herein, the reference period could affect the expected death count and, therefore, the excess death count. For instance, the reference period of Zhang et al. (2024) was 16 December 2019–6 December 2022 [20]. Most of this period was when the Zero COVID policy was implemented. In contrast, Huang et al. used 2018–19 as the reference period and found a death deficit (i.e., observed < expected) of 1,480,900 from January 2020 to November 2022 [16]. In other words, using the pre-pandemic time period as the reference period could give a higher expected death estimate and, therefore, a lower excess death estimate. How far into history one should go before the pandemic as the baseline to constitute the expected value may not have a universally accepted answer. Meanwhile, Raphson and Lipsitch’s reference period was five consecutive December–January seasons in December 2017–January 2022, spanning from the pre-pandemic era to the Zero COVID policy period. Their rationale was that the death rate remained the same during the early phase of the pandemic, and no other nationwide disaster would have caused abnormal mortality [11]. By only using data from the two winter months, their study could avoid the potential confounding factor of seasonality, should mortality data in China follow a seasonal pattern. In contrast, the other four English studies did not account for seasonality.

Both Chinese studies provided age and sex-stratified excess death estimates, as Zhang et al. analysed official death records [20, 21]. Zhang et al. (2024) also provided excess death estimates stratified by chronic condition [20]. Adjusting for population structure in age, sex, and other risk factors that are known to be associated with mortality risk and COVID-19 mortality risk in particular could help generate more accurate estimates. However, the other studies included herein were limited by the available data. If the proportion of deaths underestimated is constant between the reference period and the COVID-19 wave period, the relative changes in mortality rate could be more accurate than the absolute numbers. The two studies using Baidu index data rested on the assumption that the correlation between online searches of mortality-related keywords and actual mortality continued to hold from the reference period to the COVID-19 wave period [16, 19].

#### Estimating actual mortality using unconventional data

Besides the uncertainty associated with the expected death counts, all but two studies reviewed herein had an additional challenge: estimating actual death counts when death registration data were not available. Large uncertainties were associated with excess mortality estimates because both actual and expected death estimates were uncertain.

Excess mortality studies that relied on unconventional data (obituaries and internet search volumes) generated estimates with wide confidence bands. Counting obituaries of university staff or senior members of the Chinese Academy of Engineering and using their mortality rates as a proxy might underestimate deaths in the at-risk population, as they receive better-than-average healthcare in China. A 2024 paper [31] found that the prepandemic death rates of the Chinese Academy of Engineering doubled that of the Chinese Academy of Sciences, highlighting the uncertainty associated with the sample choice in Raphson and Lipsitch [11]. The same paper suggested that while obituary data of elite academician cohorts were likely accurate, obituary collections of university staff might suffer from missing data [31].

Baidu search volume depends on three variables: internet penetration, internet search habits of users, and the proportion of users choosing Baidu as their search engine. Lower-than-average access to the internet among the lowest socioeconomic stratum of Chinese society may underestimate their mortality. Internet search habits and Baidu market shares vary over time. For example, the market share of Baidu among all search engines in mainland China was 60.87% in December 2022 and 65.21% in January 2023 [32]. Such variation could bias the mortality estimates upwards or downwards.

#### All-cause mortality and COVID-19 deaths

Excess mortality studies estimated the all-cause mortality attributable to the COVID-19 wave, including deaths not directly caused by COVID-19. Therefore, their death count estimates (Jha et al., 2.7 million [17]; Huang et al., 2.9 million [16]; Xiao et al., 3.2 million (≥30 years old) [19]) would be higher than those provided by studies that estimated infection counts and then applied IFR to estimate deaths directly caused by SARS-CoV-2 infection only (Amemiya et al., 1.44 million [22]; Cheng et al., 1.18 million [23]; Du et al., 1.41 million [9]; Ioannidis et al., 153–249 thousand [24]; Cai et al., 1.55 million [25]; Leung et al., 0.80–1.09 million [26]; see Supplementary Texts S2 and S3).

Age-dependent risk of death, both IFR and all-cause mortality risk, could be a major source of uncertainty. IFR is difficult to ascertain, given the challenge of ascertaining the true number of infections in the community. Modelling studies (Supplementary Texts S2 and S3) relied on IFR data from other jurisdictions, such as Hong Kong or South Korea. This may underestimate the COVID-19 mortality risk in some of the less developed parts of China, where healthcare is less accessible. Meanwhile, delineating age groups, especially among the elderly, also plays a role here. The mortality risk increases sharply as one progresses into the very old age (e.g., ≥80). If older adults are grouped into larger age bins, e.g., ≥65 or ≥70, both the IFR and the all-cause mortality risk of the age group would be lower on average, underestimating the number of deaths of the very old. Given the age-dependent risk of COVID-19-associated mortality, more granular data among the very old will be beneficial in both data analysis and modelling to obtain a more accurate estimate. Further discussion on the six studies reviewed in Supplementary Texts S2 and S3 is provided in Supplementary Text S4. Discussion on the importance of the COVID-19 wave after the end of the Zero COVID policy in China in the history of public health can be found in Supplementary Text S5.

### Significance of this study

This systematic review summarized excess death estimates in mainland China in the COVID-19 wave after the Zero COVID policy ended. The unavailability of nationally registered death data made excess death estimation a double challenge: estimation of both total and expected deaths. This systematic review provided a detailed analysis of the data used in each study and the sources of uncertainty associated with their estimates, and therefore, provides a solid foundation for future studies of excess deaths in similar circumstances worldwide.

### Limitations

Limitations of this systematic review are as follows. We did not register this systematic review, and we did not publish a protocol prior to the review process. We did not create a separate data collection form; the data collected are presented in the various tables in this systematic review. The risk of bias assessment of included studies was not part of the original plan. We decided to apply a modified NOS to conduct a risk of bias assessment after the first draft of the systematic review was written. No meta-analyses were conducted because the methods used in the included studies were very different from each other.

This systematic review highlighted the differences in methods, assumptions, and data sources that could be the sources of uncertainty in the excess mortality estimates. A lack of common reference national data highlighted the need for caution when interpreting estimates derived using varied methodologies.

## Conclusions

This systematic review summarizes studies that estimated excess deaths in mainland China during the COVID-19 wave after the Zero COVID policy ended on 7 December 2022. Two Chinese papers provided excess mortality rate estimates of 153.6% and 174.3%, respectively, analysing official death records of parts of Shanghai. The excess mortality rate estimates by the five English papers vary greatly (ranging from 2.69% to 767%), given their various estimates of total and expected death counts. The estimated national death count ranged from 2.70 to 3.17 million, and excess deaths ranged from 0.71 to 1.87 million, depending on assumptions and data sources used. The total death counts estimated by these excess mortality studies were higher than six other studies that estimated COVID-19 deaths (but not excess deaths), which estimated 249 thousand to 1.55 million deaths in total. This systematic review highlights the methodological heterogeneity across studies estimating excess deaths in this setting, resulting in substantial variability and uncertainty in the reported estimates.

## Supporting information

10.1017/S0950268826101022.sm001 Fung et al. supplementary material 1 Fung et al. supplementary material

10.1017/S0950268826101022.sm002 Fung et al. supplementary material 2 Fung et al. supplementary material

## Data Availability

The data that support the findings of this review are presented in the tables and the supplementary materials.

## List of abbreviations

China CDC: Chinese Center for Disease Control and Prevention
CI: confidence interval
COVID-19: coronavirus disease 2019
CrI: credible interval
IFR: infection-fatality ratio
NOS: Newcastle-Ottawa Scale
PRISMA: Preferred Reporting Items for Systematic Reviews and Meta-Analyses
SARS-CoV-2: severe acute respiratory syndrome coronavirus 2
WoS: Web of Science Core Collection

## Supplementary material

The supplementary material for this article can be found at http://doi.org/10.1017/S0950268826101022.
