# Supplementary material for: Excess mortality in Mainland China after the end of the Zero COVID policy: A systematic review
Source: Epidemiol Infect. 2026 Feb 6;154:e29. doi: 10.1017/S0950268826101022 (PMC12964144; doi:10.1017/S0950268826101022)
Supplement: Fung et al. supplementary material 2 — Fung et al. supplementary material [file S0950268826101022sup002.docx]

**Supplementary materials for: Excess Mortality in Mainland China after the End of the Zero COVID Policy: A Systematic Review**

Contents

[Supplementary Text S1 2](#_Toc208174801)

[Supplementary methods 2](#_Toc208174802)

[Systematic literature search 2](#_Toc208174803)

[Inclusion and exclusion criteria 4](#_Toc208174804)

[Screening, review, and data extraction 4](#_Toc208174805)

[Risk of bias assessment 7](#_Toc208174806)

[Applying age-specific mortality ratios from Zhang et al. (2024) to the entire mainland China 7](#_Toc208174807)

[Supplementary results 7](#_Toc208174808)

[Risk of bias assessment 7](#_Toc208174809)

[Chinese articles: Official death record data from Shanghai 8](#_Toc208174810)

[English articles 12](#_Toc208174811)

[Supplementary Text S2 19](#_Toc208174812)

[Final size equation with vaccination-reduced susceptibility 19](#_Toc208174813)

[Individual-based transmission model 20](#_Toc208174814)

[Individual-based model using China CDC sentinel surveillance data 23](#_Toc208174815)

[Applying Hong Kong and South Korea IFR to mainland China 26](#_Toc208174816)

[Supplementary Text S3 28](#_Toc208174817)

[Scenario analysis by the Shanghai team in 2022 28](#_Toc208174818)

[Scenario analysis by the Hong Kong team in 2022 30](#_Toc208174819)

[Supplementary Text S4: 33](#_Toc208174820)

[Discussion of the six studies reviewed in Supplementary Texts S2 and S3 33](#_Toc208174821)

[Supplementary Text S5 34](#_Toc208174822)

[Importance in the history of public health 34](#_Toc208174823)

[References 35](#_Toc208174824)

[Supplementary Tables 40](#_Toc208174825)

# Supplementary Text S1

## Supplementary methods

### Systematic literature search

We limited our search to publications published on or after 1 January 2023, as we intended to only include articles that studied the phenomenon during or after it happened and excluded articles of scenario analysis anticipating the opening up of mainland China before the decision to end the Zero COVID policy was made. Operationally, we applied the same range of publication dates across all database searches: 2023/01/01 to 3000/12/12. (Note: When we applied a custom date range in PubMed, and we only specified the start date and not the end date, PubMed would automatically use 12 December 3000 as the end date. We therefore applied the same end date to the other databases.)

We applied the same set of keyword combinations to both English-language databases. The difference was in database-specific syntax:

1. PubMed (search performed on 2024-05-09 at 10.49 am EDT):
   - All Fields: (((COVID-19) OR (SARS-CoV-2)) AND (China)) AND ((Excess Death*) OR (Excess Mortality))
2. Web of Science Core Collection (search performed on 2024-05-09 at 11.18 am EDT):
   - All Fields: (((ALL=(COVID-19)) OR ALL=(SARS-CoV-2)) AND ALL=(China)) AND (ALL=(Excess Death*) OR ALL=(Excess Mortality))

The same keyword combination was translated into Chinese and was applied across all 5 Chinese-language databases. For COVID-19, we also add the term 新冠Xīnguān. Xīnguān is the short form of “new coronavirus” and has been routinely used in Chinese as the most commonly used name for COVID-19. For the keyword China, we use (中国 OR 我国), aka “China OR our country,” because in many Chinese-language articles, the authors wrote “our country” in place of China.

Keywords: (新冠 OR (COVID-19) OR (SARS-CoV-2)) AND (中国 OR 我国) AND (超额死亡)

1. CNKI (China National Knowledge Infrastructure) (search performed on 2024-05-16 at 11:26 am GMT+8)
2. Wanfang (search performed on 2024-05-16 at 11:32 am GMT+8)
3. Sinomed (search performed on 2024-05-16 at 11:36 am GMT+8) (<http://www.sinomed.ac.cn//index.jsp>)
4. Chinese Medical Journal Full Text database (search performed on 2024-05-16 at 1:28 am GMT+8) (<https://www.yiigle.com/index>)
5. Chaoxing (search performed on 2024-05-16 at 1:15 am GMT+8) (<https://qikan.chaoxing.com/>)

We chose the publication date starting from 1 January 2023 as a trade-off between sensitivity and specificity. Even researchers who studied the pertinent COVID-19 wave in real-time would have their paper published in academic journals in 2023, given the time required for peer review. The two 2022 *before*-the-fact scenario analyses that were often cited by the papers reviewed herein will be discussed in Supplementary Text S3.

We repeated our search in 2025 with the exact search terms for publications published since our last search:

1. PubMed (search performed on 2025-07-27 at 9.51am EDT): Publication Date: From 2024-05-09 to 3000-12-12
2. Web of Science Core Collection (search performed on 2025-07-27 at 10.21am EDT): Publication Date: From 2024-05-09 to 3000-12-12
3. CNKI (search performed on 2025-08-03 at 2.15am GMT+8): Publication Date: From 2024-05-16 to 3000-12-12
4. Wanfang (search performed on 2025-07-31 at 11.27pm GMT+8): Publication Date: From 2024-01-01 to 3000-12-12
5. Sinomed (search performed on 2025-08-01 at 0:21am GMT+8): Publication Date: From 2024-01-01 to 3000-12-12
6. Chinese Medical Journal Full Text database (search performed on 2025-07-31 at 11.39pm GMT+8): Publication Date: 2024-05-16 to 3000-12-12
7. Chaoxing (search performed on 2025-08-02 at 1:15am GMT+8): Publication Date: From 2024-01-01 to 3000-12-12

### Inclusion and exclusion criteria

We present our inclusion and exclusion criteria in Supplementary Table S1. We limited our scope to papers about (all or any parts of) mainland China because the special administrative regions of Hong Kong and Macao followed different public health policies, and their pandemic experiences differed. All included papers studied the COVID-19 wave that immediately followed the end of the Zero COVID policy on 7 December 2022, and we operationally defined that as the time period from 7 December 2022 to 31 March 2023. All included papers must estimate excess deaths (excess mortality) at the population level due to the COVID-19 wave following the end of the Zero COVID policy. We included both Chinese and English papers. No papers in other languages were found in our literature search. Only peer-reviewed research articles are included in this systematic review (Supplementary Table S1).

### Screening, review, and data extraction

Two co-authors independently performed title/abstract screening, full-text review, and data extraction (First search (2024): Chinese papers: first and co-first authors; English papers: first and second authors; second search (2025): Chinese papers: first and co-first authors; English papers: eighth and ninth authors for screening; first and eighth authors for full-text review).

In our first search (2024), after removing duplicates, the titles and abstracts of eight unique Chinese entries and 1148 unique English entries were screened. Six Chinese entries were excluded because they were unrelated to the topic (n=4) or the relevant time period was not covered in the studies (n=2). Among the English entries, 1130 were excluded, of which 1117 were unrelated to the topic. The other 13 had specific exclusion reasons, and a paper could have ≥2 reasons: the studies did not cover mainland China (n=3); the relevant time period was not covered in the studies (n=7); the studies did not estimate excess deaths (n=7); and four were not research articles (n=4) (Figure 1). The kappa for the inclusion/exclusion decision was 1. For the abstracts of the Chinese papers, the first and co-first authors reviewed each item independently and agreed on their decisions to include/exclude. For the abstracts of the English papers, the first and second authors reviewed each item independently, discussed each item, and reached an agreement on the decision to include/exclude.

After title/abstract screening, the full text of two Chinese articles and 18 English articles were reviewed. One additional Chinese article was identified from a media report [1]. After excluding one Chinese article and 13 English articles that did not meet the inclusion criteria, two Chinese [2; 3] and five English articles [4-8] were included in this systematic review. The reasons for excluding the 14 articles after full-text review are listed in Supplementary Table S2. The first and co-first authors did the full-text review of Chinese papers independently and agreed on the decision to include/exclude. The first and second authors did the full-text review of English papers independently and agreed on the decision to include/exclude; when they failed to reach an agreement over a paper, they consulted the co-first author and the senior corresponding author for adjudication and reached an agreement through consensus.

In our second search (2025), after removing duplicates, the title and abstract of one unique Chinese entry, and 926 unique English entries were screened. Of the 926 English entries, 924 were excluded. Of these excluded entries, 805 were unrelated to the topic, the other 119 articles were excluded for the following reasons (each item might have more than one reason): the study did not cover mainland China (n=49), the study did not cover the relevant time period (n=17); the study did not estimate excess deaths at the population level (n=54); the study was not a research article (n=21). One Chinese article and two English articles were retrieved for full-text review, and they were excluded afterwards. The Chinese article was excluded as it was unrelated to the topic. Of the two English articles, one did not cover mainland China; the other did not include an estimation of excess deaths at the population level (Supplementary Table S2).

The first author first reviewed all included papers, extracted data and presented them in text and tables. The co-first and second authors then duplicated the process by independently reviewing the papers. The co-first author double-checked all data presented in the manuscript draft, including the tables. The second author extracted relevant data into an independent document and repeated all the tables created by the first author. If any discrepancies were identified, the first, co-first, and second authors discussed them and reached a consensus. The senior corresponding author was asked to review the paper and adjudicate when there were disagreements between the first, co-first, and second authors. The final decision was reached by consensus.

The collected data include the reference period, the time period for which excess deaths were estimated, data sources, modeling methods of the reference period, whether seasonality, age, and sex were considered, the language of the articles, the location and population of the excess death estimates, the reported or estimated number of deaths, the expected number of deaths, the number of excess deaths (or excess mortality rate), and whether subnational excess mortality estimates were provided (Tables 1 and 2).

### Risk of bias assessment

Supplementary Table S3 presents the details of the modified Newcastle-Ottawa Scale used in this systematic review. A team of researchers at the University of Newcastle, Australia, and the University of Ottawa, Canada, proposed the original Newcastle-Ottawa Scale to assess the quality of non-randomized studies in meta-analyses [9]. Other researchers have modified the scale for prevalence studies, such as Mata et al. [10]. We further modified the scale to meet the risk of bias assessment needs of this specific systematic review. Two coauthors independently scored each of the included studies. The first and co-first authors scored the two Chinese papers. The first and second authors scored the five English papers.

### Applying age-specific mortality ratios from Zhang et al. (2024) to the entire mainland China

In addition, we extrapolated the excess mortality results of Zhang et al. (2024) [2] from an unnamed district in Shanghai to the population of mainland China, adjusting for differences in age structure through age-standardization using demographic data of the 7^th^ National Population Census of China in 2020 [11; 12]. We assumed that the district was Pudong New District (the district with the highest population size in Shanghai) to arrive at some conservative estimates. We did not extrapolate the results of Zhang et al. (2023) [3] because the age structure data of the unnamed township was not available.

## Supplementary results

### Risk of bias assessment

Supplementary Table S4 presents the results of our risk of bias assessment of the seven included studies.

### Chinese articles: Official death record data from Shanghai

Shanghai is a direct-administered municipality in eastern China. Its population size was around 24.87 million throughout the pandemic (2020‒2023) [13; 14]. Shanghai is divided into 16 second-level administrative units known as *qū* (districts), which are subdivided into multiple third-level administrative units, known as *jiēdào* (subdistricts) or *zhèn* (towns).

Two papers, written by the same first author, analyzed the officially registered death records of a town and a district of Shanghai, respectively [2; 3].

#### A town in Shanghai

Zhang et al. (2023) analyzed official death records of an unnamed town of 66,703 people (population size based on official household registration records) (Tables 1, 2) [3]. The authors identified 4,519 deaths from December 2015 to January 2023 (inclusive). December 2015 through November 2022 was defined as the baseline and the two months of December 2022 and January 2023 were the time period under study. The expected number of deaths over two months was estimated to be 125 (95% Confidence Interval [CI], 103-148) using a time series model with simplified periodicity. The actual death count was 317, with 192 (95% CI, 169-214) of these being excess deaths. The excess mortality rate was 153.60% (192/125) with 95% CI, 114.19% (169/148) to 207.77% (214/103). The authors analyzed data by month, including the first week of December 2022, as part of the time period under study, and they considered it a limitation of their study [3], which might have negatively biased estimates of impact due to re-opening. Meanwhile, the death count in February 2023 was similar to the monthly death count in the baseline period. The authors suggested that the excess mortality after the end of the Zero COVID policy “explained COVID-19’s substantial danger to people’s lives and health, and further explained why it was absolutely necessary for our country [China] to implement the Zero COVID policy for 3 years” [3].

#### A district in Shanghai

Zhang et al. (2024) analyzed the official death records in an unnamed district in Shanghai (Tables 1, 2) [2]. Data from 16 December 2019 through 6 December 2022 was used as the baseline. The time period for excess mortality estimates was from 7 December 2022 through 23 February 2023. This district had a monthly death count of about 2,700 in the baseline period, with the exception of January 2021 (n=3081) and April 2022 (n=4368) [2]. There was a sharp increase in the death count in December 2022 and January 2023. By February 2023, the death count was back to the baseline level. From 7 December 2022 through 23 February 2023, there were 20,990 reported deaths [2]. The authors estimated the expected number of deaths as 7651.39 and the number of excess deaths as 13,338.61, and therefore, the excess mortality rate was 174.33% (13338.61/7651.39) [2]. The authors also analyzed the death records by the cause of death, with the highest excess mortality rate among deaths by diabetes (367.52%), pulmonary infection (334.61%), and hypertension (274.01%) (Supplementary Table S5) [2].

#### Accessibility of Zhang et al. (2023) and (2024)

We note that Zhang et al. (2023) was published in *Shanghai Journal of Preventive Medicine* in October 2023 [3]. The finding of this paper was reported by émigré Chinese media on 23 January 2024 [1]. The paper was later taken offline in 2024.

Zhang et al. (2024) was originally published on 2 January 2024 as an Online First paper in *Shanghai Journal of Preventive Medicine* [2]. However, it was later taken offline, and the URL link no longer worked. It was not incorporated into any subsequent issues in 2024 (as of 27 June 2024, the 4 issues from January to April 2024 were available online). We could not locate any entry of Zhang et al. (2024) [2] in the five Chinese language bibliographic databases we searched. We first became aware of this paper because émigré Chinese media reported its findings on 23 January 2024 [1].

The authors of this systematic review have saved the PDF copy of the published version of both Zhang et al. (2023) and (2024) [2; 3]. Our review of these papers was based on their full texts.

#### Re-calculation of excess mortality rate in Zhang et al. (2024)

Supplementary Table S5 presents the expected, actual, and excess mortality data by sex, age group, and chronic diseases from the original Table 2 of Zhang et al. (2024) [2]. We recalculated the excess mortality rate because there were typos, formatting misalignments, or calculation errors in some excess mortality rates as printed in the original paper.

#### Applying age-specific mortality ratios from Zhang et al. (2024) to the entire mainland China

Zhang et al. (2024) reported in the Discussion section of their paper that after age-standardization as per the proportion of the population who were >60 years of age according to the 7^th^ National Population Census of 2020 [11; 12], their estimated number of excess deaths nationwide was 1,396,780 [2]. However, Zhang et al. did not show the details of how they did their calculation and did not report this number as part of their results; therefore, we did not treat their estimated number of nationwide excess deaths as their results.

Here, we recalculated the nationwide excess deaths using the actual, expected, and excess deaths by age group reported by Zhang et al. (2024) [2]. We performed age-standardization, assuming the age-specific mortality ratios Zhang et al. (2024) [2] reported in their unnamed district in Shanghai (Supplementary Table S5) could be applied across mainland China. We assumed their data were from the Pudong New District for two reasons. First, Zhang and their coauthors were affiliated with institutions of Pudong New District [2]. Second, of all districts in Shanghai, Pudong New District had the largest population size as per China’s 2020 census [11; 12]. If the population size of any other Shanghai districts were to be used instead, the mortality ratios would have been higher. In other words, we chose the most conservative estimates. We applied the demographic data from the same census that Zhang et al. (2024) used [2]: Demographic data of Pudong New District [12] and mainland China [11] were those of the 7^th^ National Population Census of China in 2020. Because the largest age group in Pudong’s demographic data was ≥85, we collapsed the 75-89 and ≥90 age groups, as found in Zhang et al. (2024) [2]. We expressed the death numbers as percentages of the population size by age group. Then, we applied these age-specific mortality ratios to the mainland Chinese population and estimated the number of all-cause, expected, and excess deaths by age group.

The all-cause mortality in the 2.5 months after the end of the Zero COVID policy (7 December 2022 – 23 February 2023) would be approximately 4.96 million if mortality rates observed by Zhang et al. (2024) [2] in a district in Shanghai were applied across mainland China. Of these 4.96 million deaths, 3.14 million would be excess deaths (Supplementary Table S6). Our recalculation of excess deaths nationwide, using the data Zhang et al. provided for the district and the same national census data that they used, arrived at an estimate that was higher than the 1.40 million reported in the Discussion section of Zhang et al. [2].

If these numbers are used as benchmarks against which other studies reviewed herein are compared, Jha et al. [5], Huang et al. [4], Raphson and Lipsitch [6], and Xiao et al. [8] (Table 2) would all have underestimated the total number of all-cause deaths in mainland China in the COVID-19 wave after the Zero COVID policy ended.

Our extrapolation is subject to limitations. Shanghai is one of the most developed cities with the best healthcare facilities in mainland China. Applying age-specific mortality ratios observed in a district in Shanghai to the entire mainland China is subject to potential bias, as the mortality situation in Shanghai was not necessarily representative of the rest of mainland China. If the mortality rate in Shanghai was lower (thanks to better healthcare in Shanghai) than in the rest of mainland China, our extrapolated estimates would *underestimate* the true extent of mortality across mainland China.

### English articles

#### Self-reported family deaths from a nationally representative sample

Jha et al. surveyed 8,004 adults (out of 210,000 panelists invited, with a 4% response rate) in 31 administrative regions of mainland China and asked about deaths in families since January 2020 [5]. Survey participants provided information about the month, year, age, and sex of the deaths in their families. The authors calculated and smoothed the age-group-specific monthly death rates using a 3-month rolling mean [5]. They found that among the age group of ≥60 years, compared with the period of January 2020 through November 2022, the average death rates had doubled during the two months of December 2022 and January 2023 [5]. They were then compared with the average national age-specific monthly mortality in China from 2012 to 2019, as reported in the United Nations Population Division’s World Population Prospects 2022. The United Nations estimated that there were 675,000 deaths per month (ignoring seasonality) among ≥60-year-olds in China in 2019. If China experienced the same mortality rate from January 2020 through November 2022, a doubled death rate would imply that mainland China experienced 1.35 million excess deaths in the 2-month COVID-19 wave at the end of the Zero COVID policy [5]. This would translate into an estimated 2.7 million deaths in total in the 2 months of December 2022 and January 2023.

However, there could be an alternative interpretation of the results of Jha et al. [5]. As presented in Figure 1 in Jha et al., the death rates of ≥60-year-olds from January 2020 through November 2022 reported in the survey were lower than those of the 2012-2019 reference period [5]. This could indicate a death deficit under the Zero COVID policy. During the COVID-19 wave of December 2022 and January 2023, the survey’s death rates were similar to those of 2012-2019. Under this alternative interpretation, there would be no excess deaths relative to the 2012-2019 reference period, as the observed was similar to the expected if COVID-19 had not occurred at all.

Prof. Jha confirmed in his personal correspondence with our team, dated 16 September 2024, that the first interpretation presented above was close to his own interpretation of his results. The emphasis was on the *doubling* of mortality rates among the ≥60-year-olds in their survey. The exact death estimates would depend on the expected value of the baseline.

There were several limitations to the survey data provided by Jha et al. [5]. Crucially, their method relied on a nationally representative sample of participants who were willing to provide accurate and truthful answers. These conditions might not have been met due to the low response rate and the high sensitivity of some questions. There could be bias in both the reported answers (social desirability bias) and among those who chose to respond (response bias), and an underestimation of deaths was expected. Indeed, as the authors suggested, “sample surveys generally underestimate deaths” [5].

The appendices of Jha et al. [5] were not available on the journal’s website. We obtained them directly from the authors via email.

#### Indirect estimation of elevated death rates using internet search volume and/or obituary data

Three papers applied innovative measures to indirectly estimate the death rates after the Zero COVID policy ended using either obituary data, internet search volume, or both. Obituary data were analyzed by two groups: Raphson and Lipsitch [6] analyzed the Chinese Academy of Engineering’s (CAE) senior members’ obituaries, while Xiao et al. [8] analyzed those of employees from three universities. Two papers analyzed Baidu Index data for the temporal trends of relative changes in internet search volume of mortality-related keywords in Baidu, mainland China's most popular search engine [15]. Supplementary Table S7 presents the terms used in Baidu Index analyses by Huang et al. [4] and Xiao et al. [8]. Huang et al. [4] analyzed a set of six Chinese keywords, while Xiao et al. [8] analyzed a set of four Chinese keywords, between which one keyword overlapped in meaning (two different Chinese keywords that have the same meaning of “cremation”). Huang et al. [4] did not present the exact Chinese search terms in their paper. They were provided to us by the authors via email (Prof Oliver Zhen Li, personal correspondence).

Raphson and Lipsitch [6] analyzed obituaries of senior (≥80-year-old) CAE members (excluding foreign members) from December 2017 to January 2023. They calculated the excess death rates of CAE members in four age groups, 80-84, 85-89, 90-94, and ≥95 year-olds, in the two months of December 2022-January 2023. The expected rates were those of the 2 months of December and January in the previous 5 winters. Among these senior CAE members, there were 26 deaths when the expected count was 3 (an excess death of 23 with an excess mortality rate of 766.67%). The authors used parametric bootstrapping to obtain the 95% confidence interval for the excess death rates. The age-group-specific excess death rates of senior CAE members were applied to the population size of the corresponding urban-dwelling ≥80-year-old men in mainland China to calculate their excess deaths by age group. The authors assumed that the risk ratio (RR) of excess deaths between <80-year-old men and ≥80-year-old men in urban mainland China was the same as the RR of COVID-19 deaths between these two age groups in Hong Kong in its 5^th^ pandemic wave (31 December 2021 – 29 January 2023). Adjusting for age structure between these two jurisdictions, the excess deaths among urban-dwelling men, in general, were estimated. The authors then assumed that the RR of excess deaths between females and males in urban mainland China was the same as the RR of COVID-19 deaths (inside and outside hospitals) between the two sexes in Hong Kong. Adjusting for the population size between sexes between urban mainland China and Hong Kong, they estimated excess deaths in urban-dwelling women in mainland China. Overall, they estimated 917,000 (95% CI, 425,000 – 1.45 million) excess deaths among urban dwellers of both sexes in mainland China [6].

Raphson and Lipsitch estimated excess deaths among urban residents in mainland China indirectly, without explicitly estimating the national number of deaths and expected deaths [6]. Their method was similar to the indirect age-standardization method. There were two key assumptions: first, the mortality risk profiles of senior CAE members were representative of urban-dwelling ≥80-year-old men in mainland China; second, the RR of excess deaths between the <80-year-old and the ≥80-year-old and that between males and females in urban mainland China, were the same as RR of COVID-19 deaths observed between similar groups in Hong Kong. The authors’ first assumption was likely to underestimate excess deaths.

Huang et al. devised a “mourning and funeral index,” which was the average of the Baidu search volume of six keywords [4]. The national “mourning and funeral index” was a population-weighted average of the 31 provincial-level indexes. The authors observed a sharp increase in this index after the end of the Zero COVID policy. Therefore, they used the monthly-mean adjusted index to extrapolate mortality through the pandemic. They used the daily average search volume in 2018 and 2019 as the base volume to calculate the aggregate excess “mourning and funeral index” for each province on each day [4]. Using pre-pandemic mortality data in 2018 and 2019 from the China Research Data Services Platform database as baseline (average annual mortality ratio multiplied by population size in each year, divided by 365), the authors calculated the expected death count by province and by day. The authors estimated each province's daily excess deaths by assuming the death count's increase or decrease was proportional to the relative increase or decrease in that province’s Baidu search volume “mourning and funeral index” [4].

Xiao et al. [8] analyzed the obituaries of employees (current and retired) of two universities in Beijing and one in Harbin, Heilongjiang province, from 1 January 2016 to 31 January 2023. The authors assumed that these universities’ employees represented individuals ≥30 years old in Beijing and Harbin, respectively. The authors applied an interrupted time-series design and included two variables in the segmented negative binomial regression models to distinguish three time periods (pre-COVID-19: January 2016 – December 2019, Zero COVID policy: January 2020 – November 2022, post-Zero COVID: December 2022 – January 2023). A strong, positive, statistically significant association existed between university-specific mortality and province-specific (Beijing and Heilongjiang) Baidu Index search volume data of 4 mortality-related keywords. The authors then extrapolated to other provinces. They assumed that the ratio between the estimated relative change in the Baidu index of the 4 keywords and the estimated relative change in the mortality rate of a specific province would be the same as that in the reference provinces (Beijing and Heilongjiang). The authors estimated the excess deaths in each province by applying the proportional increase in mortality to each region’s expected death count. The authors wrote that they derived the expected number of deaths by region and month from the 2020 China Census data and China National Disease Surveillance Points (aka Sites) without providing further details [8].

Both Huang et al. and Xiao et al. used relative changes in internet (Baidu) search volumes of mortality-related keywords as a proxy for the relative changes in mortality rates [4; 8]. Huang et al. [4] validated their method by first demonstrating a statistically significant correlation between the relative search volume of keywords and death counts in 2011-2019 (average of 6 terms, r=0.578, p<.01). They then demonstrated out-of-sample validity by building a regression model using 2011-2017 data and then compared the model prediction with the actual death count in 2018-2019. In contrast, Xiao et al. further validated their method by demonstrating the strong, statistically significant correlation between relative search volumes of 4 keywords and publicly available obituaries of faculty and staff of 3 universities in mainland China (Beijing, r=0.95, p<.001; Heilongjiang, r=0.97, p<.001) [8]. Huang et al. derived their expected death count using the 2018-2019 mortality data. However, they did not report their expected death count in the paper [4] (available from the authors through personal communication). In contrast, Xiao et al. derived their expected death count from 2020 census data and China National Disease Surveillance Points [8].

The use of obituary data in Raphson and Lipsitch [6] and Xiao et al. [8] raised the question of potential bias, as the sample (CAE senior members or university faculty/staff, respectively) might not be representative of the population. A 2024 paper by Ioannidis [16] found that the pandemic death rates of CAE members doubled that of the Chinese Academy of Sciences, highlighting the uncertainty associated with the sample choice in Raphson and Lipsitch [6]. Prepandemic death rates of CAE members were 4 to 12-fold lower than those of their respective population age strata in mainland China, suggesting that CAE members were not representative of the general population as far as death rates are concerned [16]. Regarding the university cohorts in Xiao et al. [8], Ioannidis [16] suggested that, besides representativeness, missingness could be a bigger problem with university cohorts than with elite academician cohorts, as it was unlikely that obituary collections covered all deaths in the university cohorts.

#### Using a linear model to estimate the expected death count in Taiyuan

Wang et al. [7] only estimated the excess deaths in Taiyuan City, Shanxi province, in 2022. The authors first estimated the population size of Taiyuan in 2022 by fitting the 2017 to 2021 data to a logistic population model. Then, they fit a linear model to the actual death numbers in Taiyuan in 2017, 2018, 2019, and 2021, excluding 2020, to estimate the death rate in these years. They then estimated the expected death number in Taiyuan in 2022 to be between 28,976 and 32,976 [7]. They wrote that it was “known” that the death count in Taiyuan in 2022 was 33,863 without a source. The range of excess death estimates in Taiyuan in 2022 was, therefore, 887 to 4,887 [7]. We highlight a few limitations of this study, given its specific assumptions. First, the excess deaths in 2022 were attributable to the COVID-19 wave after the end of the Zero COVID policy. Seasonality of mortality data was not considered. Second, the expected death count depended on the estimated mortality rate of previous years (2017-19, 2021) using a linear model and the estimated population size in 2022 using a logistic growth model [7]. If the authors excluded the 2021 data point from their linear model, they would have fewer expected deaths and more excess deaths. It is also unclear if a logistic growth model of population growth approximates the reality. Third, we note that in the first part of the study, the authors used a dynamic model to estimate the infection count of the COVID-19 wave and estimated that 85% of the Taiyuan population was infected by 28 December 2022. However, they did *not* use the infection count to estimate the death count in the second part of their study. The two parts were disconnected.

# Supplementary Text S2

Four papers excluded after a full-text review estimated the COVID-19 mortality, but not excess mortality, after the end of the Zero COVID policy [17-20]. They are reviewed in Supplementary Text S2 so that we would have a more comprehensive view of the probable size of the pandemic wave and the associated COVID-19 death count. Their methods, data, estimated death counts, and selected sensitivity/scenario analyses are summarized in Supplementary Tables S8 and S9, respectively.

## Final size equation with vaccination-reduced susceptibility

Amemiya et al. [17] used an age-structured Susceptible-Infectious-Recovered (SIR) model-derived final size equation with vaccination-reduced susceptibility accounted for to estimate the number of infected people in the pandemic wave immediately after the end of the Zero COVID policy. The authors then applied the age-specific infection-fatality ratio (IFR) to calculate the mortality.

The authors’ age-structured model grouped people into 10-year age groups (with ≥80-year-olds as one group) (Supplementary Table S10). Given that most people in mainland China received the inactivated COVID-19 vaccine, the authors’ model [17] accounted for their protection against infection with the Omicron variant, with people who had only received the first dose not protected at all. A second dose of inactivated vaccine was assumed to protect vaccinees against infection at 0%, 10%, or 20% (sensitivity analysis) and against death at 90.3% [21]. A third dose of inactivated vaccine was assumed to protect vaccinees against infection at 33.4% [22], and against deaths at 91.1% [23].

The contact matrix in their age-structured model used data from Japan [24], assuming the social contact pattern was the same between mainland China and Japan. The age-specific IFR was obtained from O’Driscoll et al. [25], which collated national-level age-specific COVID-19-related death counts from 45 countries, including China, in 2020 (Supplementary Table S11).

In their main analysis, the authors assumed an R_0_ of 3.4 (the same as in the R_t_ estimated in Shanghai in the first week of March 2022 in Cai et al. [26]). They estimated that 1.4 million people died (half of whom were ≥80 years old) in the pandemic wave after the end of the Zero COVID policy [17].

The authors performed a sensitivity analysis with R_0_ ranging from 1 to 10. They also analyzed if the 3^rd^-dose coverage was 10 or 20% lower or higher than the actual value for all age groups. They found that assuming a 20% 2^nd^-dose effectiveness, a random increase in the 3^rd^-dose coverage by 10% and 20% would prevent 16,367 and 33,228 deaths, respectively [17] (Supplementary Table S9).

The authors also performed a scenario analysis in which the BioNTech mRNA vaccine (BNT162b2) was used instead of inactivated vaccines, with R_0_ ranging from 1 to 10. The authors found that if an mRNA vaccine was used instead of inactivated vaccines, the estimated death count would be 1.1 million. Similar to the main analysis when the inactivated vaccine was used (in reality), in the mRNA vaccine scenario, about half of all deaths were ≥80 years old, and 20% of all deaths were 70-79 years old [17] (Supplementary Table S9).

Amemiya et al. [17] provided us with their supplementary tables through emails as they were unavailable from the journal website when writing this systematic review.

## Individual-based transmission model

Cheng et al. [18] asked the counterfactual question: What could China have done better when it ended the Zero COVID policy? Would a “safe exit” be possible? Cheng et al. [18] defined a “safe exit” from the Zero COVID policy as having mortality rates lower than 14.3 per 100,000 persons (the influenza mortality rate in mainland China). They explored the intervention parameter combinations that made safe exits possible. Our reading of this paper suggests that the worst-case scenario in this paper could be taken as their estimation of the reality in mainland China after the end of the Zero COVID policy. The median mortality rate in the baseline worst-case scenario was 83.7 per 100,000 persons [18]. Assuming a population size of 1,409,778,724 in mainland China as in Du et al. [19], the mortality rate of Cheng et al. [18] would translate into an estimate of 1,179,985 deaths as a result of the COVID-19 wave after the Zero COVID policy ended.

Cheng et al. [18] used an individual-based transmission model of a synthetic population of 500,000 to simulate COVID-19 transmission in mainland China at large and 3 representative cities, Shanghai (eastern China), Shenzhen (southern China), and Shiyan (central China), with different healthcare capacities, age structure, and age-specific vaccine coverage. The authors assigned individuals in the model to 16 age groups (Supplementary Table S12) and 4 vaccine dose groups (0 to 3 doses) given the age structure and the age-specific dose-specific coverage of the location. The age-structured contact matrices used (*all* and only *home* settings) were from a study in Shanghai in 2017 [27]. The assumption was that people resumed their pre-pandemic life and work routines immediately as the Zero COVID policy ended. The authors assumed that when a positive case patient was identified, they would self-isolate at home, and their contact with family members would be 20% of the average in the *home* setting. One of their sensitivity analyses was what if there was no reduction in contact rates at home [18].

The model assumed 9 states in disease progression. A susceptible individual, upon infection, would enter the “exposed” state (latent period). The individual could become either asymptomatic and infectious or presymptomatic and infectious. The presymptomatic individual would become mildly symptomatic and infectious. Both the asymptomatic and the mildly symptomatic individuals could recover. The mildly symptomatic individual could also become severely ill or critically ill. The severely and critically ill would be isolated and, therefore, not infectious. They could either recover or die [18].

Cheng et al. assumed that 1.5% of asymptomatic and presymptomatic cases and 75% of symptomatic cases would be detected through full coverage of rapid antigen tests with a 75% sensitivity [18].

Cheng et al. assumed that 0.6 cases were imported to the synthetic population each day based on the per-capita importation rate to Hubei province in early December 2022 [18].

For each set of parameter values, they simulated 48 times to obtain the median estimated mortality rate. In their base case scenario, they assumed a mask coverage of 75% [18].

Cheng et al. validated their model by comparing their simulated IFR results with those of the observed IFR data from Shanghai in Spring 2022 and found them comparable [18].

Cheng et al. [18] explored combinations of interventions. They explored a range from 0 to 100% of the population wearing masks. They also explored the number of ICU and hospital beds per capita in a range from their current value in the specific location to the worldwide maximum of 48 ICU beds per 100,000 and 14.4 hospital beds per 1000. Cheng et al. [18] assumed that if no hospital beds were available for severe case patients, they would stay home and experience 10 times the mortality rates than those hospitalized. For the critical case patients, if no ICU beds were available to them, they would surely die. Cheng et al. [18] assumed the 1^st^, 2^nd^, and booster doses of the inactivated vaccine reduced susceptibility to infection by 3.1%, 7.0%, and 13.1%, and reduced the overall probability of death by 54.6%, 74.8%, and 88.8%, respectively. They assumed a constant rate of immunity waning until a stable rate was reached. They also assumed that 1^st^, 2^nd^, and booster doses could reduce the probability of onward transmission by 0%, 0%, and 5.3%. Cheng et al. [18] explored increasing vaccination coverage by having 1 additional dose among different age groups (ranging from the proportion of 0 to 1). Cheng et al. [18] also explored the possibility of prescribing antiviral therapy to individuals who were ≥12 years old that would reduce the rate of hospitalization and death by ~80%. They set it to a range from the proportion of 0 to 1 among the eligible age groups [18].

Interested readers may refer to Cheng et al. [18] for the detailed findings of their many scenario analyses. Here, we only highlight their baseline scenario, where the *average* mortality rate was 29.3 per 100,000 persons nationally [18]. This translated into a death count of 413,065. This was only possible given Cheng et al.’s scenarios of what China could have done better. We note that the estimated death count in the baseline *worst-case* scenario of Cheng et al. [18] (~1.1 million) was lower than the estimates of ~1.4 million in Amemiya et al. [17] and Du et al. [19]. This was likely to do with the specified values of the age-group-specific parameters of the proportion of asymptomatic cases, the probability of becoming a severe case from a mild case, the probability of becoming a critical case from a mild case, the probability of death from a severe case, and the probability of death from a critical case. We calculated the age-group-specific IFR in Supplementary Table S12 based on these parameter values. We note that the IFR for the ≥75-year-olds was much lower in Cheng et al. [18] than the IFR for the ≥80-year-olds in Amemiya et al. [17] and Du et al. [19]. This indicates the importance of having more granular data for the older age groups, which could eventually affect the mortality calculation.

## Individual-based model using China CDC sentinel surveillance data

Unlike Cheng et al. [18], Du et al. [19] was not a mechanistic transmission model. Du et al. incorporated the data from the Chinese Center for Disease Control and Prevention (China CDC) sentinel surveillance’s daily test positivity reports into a stochastic individual-based model [19]. They estimated the number of deaths in mainland China among people who would have been infected from 8 December 2022 through 19 January 2023. The authors assumed that the daily test positivity reports from 16 December 2022 to 19 January 2023 were accurate. They took the mean IFR data from Leung, Leung, and Wu [28], and used the ratios of lower and upper CIs to the mean in the data from the paper by the COVID-19 Forecasting Team [29] to scale the 95% CI (Supplementary Table S11). Their vaccination-related data was based on Cai et al. [26].

Du’s model was a synthetic population of 1 million with designated age per mainland China's national age distribution (Supplementary Table S10) [19]. Their model was run 1000 times to obtain the median and the 95% credible intervals (CrI). In each simulation, the age-group-specific IFRs were sampled from their distributions and were assigned to each individual their age-group-specific IFR. Du et al. [19] then randomly selected a number of people who would test positive for the first time on a given day, as per the China CDC sentinel surveillance system’s reported SARS-CoV-2 test positivity for each day from 16 December 2022 through 19 January 2023. The authors estimated the CI for the reported test positivity, given a sample size of 2500, and then drew a random number from the normally distributed sampling distribution for the test positivity statistic. Then, the authors multiplied the number by 1 million. For each individual in the model, their date of infection was then determined, with the earliest possible date of 8 December 2022. This was done by first identifying the time difference between 7 December 2022 and the day the individual tested positive and assuming that in the prior sampling periods, they tested negative (and a random date was chosen for each sampling period). Then, the authors determined the probability of having been infected on a given day, given negative tests on those prior test dates and a positive test on the day the person tested positive. An infection date was then assigned randomly according to this probability distribution. The authors then assigned each positive individual in the model a vaccination history, as per the reported daily age-specific vaccination rates in mainland China, as in Cai et al. [26]. The vaccine-induced protection against death was assigned to each positive individual, assuming protection began 2 weeks after each dose and immunity waned stepwise 6 months following each dose. The authors then determined if the individual would die from COVID-19, given their IFR and vaccine-induced protection. If so, they would randomly select a date of symptom onset as per the incubation period distribution and then randomly select the date of death as per the estimated distribution of the lag from onset of symptoms to death. The authors finally scaled up the simulated results from 1 million simulated individuals to mainland China’s entire population by age group [19].

Given that about nine-in-ten people in mainland China were infected in that 35-day period as per China CDC’s sentinel surveillance, Du et al. found that approximately 1.41 million (95% CrI, 1.14-1.73 million) people died in the pandemic wave after the end of the Zero COVID policy. Of these deaths, 0.80 million (95% CrI, 0.60-1.05 million) were of adults >80 years old [19].

Du et al. [19] conducted 6 sensitivity/scenario analyses. First, the Ineffective Vaccine Scenario (S1): If all primary and booster doses were of 0% vaccine effectiveness, 5.11 million (95% CrI, 4.15-6.28 million) people would have died, of whom 2.93 million (95% CrI, 2.16-3.91 million) were >80 years old. Second, the Durable Vaccine Scenario (S2): If vaccine effectiveness did not decline after 6 months, 1.32 million (95% CrI, 1.06-1.62 million) people would have died, of whom 0.76 million (95% CrI, 0.55-1.01 million) were >80 years old. Third, the Hospital Surge Scenario (S3): If the age-group-specific IFR increased 3.39-fold during the pandemic peak in mainland China (December 20-22, 2022), the same magnitude in IFR increase in Hong Kong in March 2022 (compared to the end of October 2022), 2.11 million (95% CrI, 1.71-2.60 million) would have died, of whom 1.21 million (95% CrI, 0.89-1.62 million) were >80 years old. In the fourth and fifth scenarios (S4&S5), the authors explored the same model but with either 2 million or 500 thousand individuals in the synthetic populations and obtained similar results to the main analysis. In the sixth scenario (S6), the authors applied an alternative set of age-specific vaccine effectiveness data drawn from McMenamin et al. [30], and found that 1.56 million (95% CrI, 1.28-1.93 million) would have died, of whom 0.90 million (95% CrI, 0.66-1.21 million) were >80 years old [19].

## Applying Hong Kong and South Korea IFR to mainland China

Ioannidis et al. conducted their study in December 2022 and projected their forecast of COVID-19 mortality in mainland China at the end of the Zero COVID policy from 7 December 2022 to summer 2023 [20]. Their study was published on 23 January 2023.

The authors estimated the age-stratified IFRs in Hong Kong and South Korea in their respective Omicron waves in 2022 (Supplementary Table S11). They extrapolated those IFRs to the population age structure of mainland China (Supplementary Table S10). In their *maximum death* scenario, the authors assumed that the *entire* population of mainland China was to be infected. Applying Hong Kong and South Korea IFRs, the maximal expected number of COVID-19 deaths in mainland China in the pandemic wave after the end of the Zero COVID policy was 987,455 and 619,549, respectively [20].

Ioannidis et al. performed sensitivity analyses, including a range of lower attack rates (25% to 70%) among the 0-to-59-year-olds and a range of public health interventions able to protect the ≥60-year-olds (from no effect to reducing fatality impact by 75%). In their main scenario, where only 45% of the 0-to-59-year-olds were infected, and the fatality impact among ≥60-year-olds was reduced by 50%, the estimated number of COVID-19 deaths was either 152,886 (South Korea IFR) or 249,094 (Hong Kong IFR), depending on the choice of estimated IFR [20].

Investigating why Ioannidis et al.’s estimates were substantially lower than those of the other papers is interesting. While Du et al. [19] utilized China CDC sentinel surveillance data and expected that 90% of the population would get infected, the maximum death scenario in Ioannidis et al. [20] assumed everyone in mainland China would get infected in the same COVID-19 wave. We suspected that the main issue was not in the estimation of infection count but in the choice of IFR and the population structure. We note that Ioannidis et al. assumed that 4 million people in mainland China resided in long-term care facilities: 1, 1, 1.5, and 0.5 million in the age groups of 60-69, 70-79, 80-89, and ≥90 year-olds, respectively. These people would have a different IFR than the elderly residing in the communities (Supplementary Table S11). Both sets of IFR (Hong Kong and South Korea) estimated by Ioannidis et al. were significantly lower than that of the IFR used in Amemiya et al. [17] and Du et al. [19], in age groups of 30-year-olds and older. The majority of deaths in Ioannidis et al. [20] would occur among the elderly residents of the long-term care facilities who were assumed to have an IFR of 9% (Hong Kong IFR) or 1.17341% (South Korea IFR). The combination of IFRs applied by Ioannidis et al. [20] and their choice of population structure assumption happened to be more optimistic than the other papers.

# Supplementary Text S3

Two studies (Cai et al. [26] and Leung, Leung, and Wu [28]) were frequently cited by papers included in our review and those in Supplementary Text S2, but they were not identified in our search, as they were released in 2022. They did not estimate excess mortality, and thus, they did not meet our inclusion criteria. However, we deemed it beneficial for our discussion to review these two studies in Supplementary Text S3 because they laid the groundwork with which subsequent research was conducted. Supplementary Tables S13-S14 summarized the methods, data sources, estimated death count in the baseline scenario, and selected sensitivity/scenario analyses.

## Scenario analysis by the Shanghai team in 2022

Cai et al. published their scenario analysis of pathways of reopening on 10 May 2022 in *Nature Medicine* (July 2022 issue), stating that mainland China could safely end its Zero COVID policy [26]. This study was done amidst the Omicron wave in Shanghai in Spring 2022, which led to Shanghai's 2-month (April-May) lockdown [31]. In the aftermath of the lockdown of Shanghai, the negative impact on the economy and, therefore, people’s livelihoods became apparent to many people both inside and outside China. As Cai et al. wrote, China was “considering whether, and for how long,” the Zero COVID policy could “remain in place” [26].

Cai et al. [26] used an age-structured stochastic compartmental model to simulate Omicron variant transmission in mainland China. The model incorporated 14 age groups with pre-COVID-19 contact matrix data from China [32]. The model accounted for asymptomatic infection, vaccination (1^st^ dose, 2^nd^ dose, booster), waning immunity, hospitalization, ICU, and antiviral therapy. In the main analysis, they considered lower susceptibility to infection among children and adolescents than adults (sensitivity analysis: same susceptibility across age groups); asymptomatic and symptomatic individuals equally infectious (sensitivity analysis: an asymptomatic individual being 65% less infectious) [26]. The proportion of symptomatic cases requiring hospitalization and ICU admission risk were age-dependent. This model *assumed all deaths occurred among hospitalized patients* [26]. Supplementary Table S15 presents the population structure of mainland China (2020) and the national vaccination coverage (as of March 2022) used to parameterize the model in Cai et al. [26]. The total population size was exactly the same as that used in Du et al. [19]. Cai et al. [26] assumed that in mainland China, there were a total of 3,143,889 hospital beds (3,080,362 non-ICU beds and 63,527 ICU beds) reserved for respiratory illness nationally.

Their age-specific IFR and infection-hospitalization risk among unvaccinated individuals were derived from the Omicron wave in Hong Kong. They derived the age-specific IFR from age-specific case-fatality ratios among unvaccinated individuals and the overall infection-reporting ratio; and the age-specific infection hospitalization risk from the age-specific IFR and the fatality risk among hospitalized patients [26].

Cai et al. adjusted the age-specific ICU admission risk of unvaccinated hospitalized patients that was associated with the ancestral strain in China [33] by comparing the overall ICU admission risk among unvaccinated hospitalized patients between the Omicron variant and the ancestral strain [26].

Based on the parameter values provided by Cai et al. [26], we calculated the IFR for the unvaccinated people in Cai et al.’s model (Supplementary Table S16) [26].

In the baseline scenario, Cai et al. [26] simulated a hypothetical pandemic wave, seeded with 20 Omicron index cases on 1 March 2022, with a reproduction number of 3.9 (such that after accounting for vaccine-conferred protection, the effective reproduction number dropped to 3.4, that was empirically estimated by the authors as the reproduction number in the early phase of the Omicron epidemic in Shanghai from 1 March to 8 March 2022). The authors assumed that booster doses were rolled out at a rate of 5 million doses per day, starting on 1 March 2022, that 90% of those eligible (completed the 2^nd^ primary dose at least 6 months prior) received the booster, that vaccine efficacy or effectiveness was similar to what was observed in Hong Kong or Brazil with a low immune escape scenario (e.g., having the booster dose of the inactivated vaccine protected vaccinees against mortality by 98.4%), and that antiviral therapies were not available. In this baseline scenario, Cai et al. found that over a 6-month period, 1.55 million deaths (1.10 per 1000), 2.66 million ICU admissions (1.89 per 1000), 5.08 million hospitalizations (3.60 per 1000), and 112.19 million symptomatic infections (79.58 per 1000) would happen as a consequence of the pandemic wave [26].

Besides the baseline scenario, Cai et al. performed extensive sensitivity/scenario analyses, including running simulations specific to 3 administrative regions (the direct-administered municipality of Shanghai and the provinces of Shanxi and Shandong, respectively), different scenarios of interventions (including different vaccination scenarios and nonpharmaceutical interventions [NPI]), different effective reproduction number, and different combinations of these scenarios [26].

With the benefit of hindsight, we now know that when the Zero COVID policy ended on 7 December 2022, none of the interventions proposed by Cai et al. [26] in summer 2022 to mitigate the impact of the anticipated pandemic wave was in place in mainland China in December 2022. Therefore, the baseline scenario of Cai et al. [26] appeared to reflect better what happened eventually. The estimated 1.55 million deaths were in the same order of magnitude as the estimates of Amemiya et al. (final size equation) [17] and Du et al. (individual-based model) [19].

## Scenario analysis by the Hong Kong team in 2022

Leung, Leung, and Wu [28] released a preprint on medRxiv on 14 December 2022, just one week after mainland China ended its Zero COVID policy.

The authors used an age-structured metapopulation compartmental model (previously used in Mistry et al. [32] and Leung, Wu, and Leung [34]), incorporated age demographics and contact patterns specific to mainland China, and adapted it for this project. This model incorporated the intra-China population movements among >300 prefecture-level cities around the Lunar New Year (known as *chunyun*). Their analysis focused on the simulation results of two megalopolises (the Yangtze River Delta Region and the Pearl River Delta Region) and two provinces with more rural populations (Henan and Guangxi) [28].

The authors simulated 5 scenarios [28]. Scenario 1 was the status quo without a 4^th^ dose vaccine, antivirals, and NPIs. Scenario 2 included mass vaccination of the 4^th^ dose vaccine and a 60% coverage of antivirals. The vaccine campaign would begin 30 days before reopening at a rate of 4% of the population per week and a maximum coverage of 85% of the population (with 80% taking “Vaccine A” and 20% taking “Vaccine B”). Scenario 3 included Scenario 2 and added NPIs that would start 14 days after the pandemic wave began, reduce R_t_ by 47% from day 15 to day 74, and gradually relax from day 75 to day 104. Scenario 4 included Scenario 3, with mass vaccination conducted at 6% of the population per week instead. Scenario 5 included Scenario 4, but the 4^th^ dose could be any one of 5 (instead of 2) brands of vaccine (equal probability) [28]. With the benefit of hindsight, Scenarios 2 to 5 never happened, and so we focused on Scenario 1 here.

The authors found that in Scenario 1, the mean cumulative incidence of deaths ranged from 568 to 770 per million [28]. If we used the same population size estimate of 1,409,778,724 as in Cai et al. [26], a rate of 568 to 770 per million is translated into a cumulative death count of 800,754 to 1,085,530 approximately.

The age-specific infection-hospitalization ratios and IFRs used in Leung, Leung, and Wu [28] are summarized in Supplementary Table S17. Leung and colleagues noted that at the peak of the pandemic wave, the hospitalization rate would be 6-7 per 10,000 persons, with a mean daily incidence of deaths of 2.3-3.5 per 100,000 persons [28]. According to the authors, in a best-case scenario, they assumed that mainland China’s healthcare capacity for COVID-19 patients would be 100% (baseline) and 200% (surge capacity) of the maximum number of beds designated for COVID-19 patients in Hong Kong in May 2022, which corresponded to 1.1 and 2.2 hospitalizations per 10,000 persons [28]. The authors assumed that IFRs would increase by 10%, 20%, 30%, 40%, and 50% when demand for hospital care outstripped supply of hospital beds by a ratio of 1 to 2, 2 to 3, 3 to 4, 4 to 5, and more than 5 to 1, respectively [28].

Leung et al. [28] stated that their assumptions were based on three pieces of information:

1. Wuhan experienced COVID-19 with IFRs ~50% higher than those experienced in provinces outside Hubei.
2. In the US, there were statistical associations between locations with smaller numbers of ICU beds, nurses, and general hospital beds per COVID-19 case and a higher number of deaths in April 2020.
3. In the US, the adjusted mortality decreased from March 2020 (25.6%) to August 2020 (7.6%), and the standardized mortality ratio decreased from March 2020 (1.26) to August 2020 (0.38).

Leung et al. [28] used China’s 2010 population census data for their age structure [35] (personal communication, Dr. Kathy Leung) (Supplementary Table S18). Given that Du et al. [19] used the same IFR values (with different 95% CI) as that of Leung et al. [28], the difference in their estimated deaths could be a result of different age structures used in the modeling (2010 data in Leung et al. [28] versus 2020 data in Du et al. [19]), and the estimated number of infections as a result of the pandemic wave. Apparently, Leung et al. [28] tended to use optimistic parameter values (as in their assumption of hospital capacity across mainland China being the same as that of Hong Kong), it is therefore understandable that their death estimates were more optimistic than those of Amemiya et al. [17] and Du et al. [19].

# Supplementary Text S4:

## Discussion of the six studies reviewed in Supplementary Texts S2 and S3

Unlike the excess mortality studies reviewed in the main text, the six studies reviewed in Supplementary Texts S2 and S3 used different statistical, mathematical, or computational models to estimate the numbers of COVID-19 infections by age group first and then applied the age-specific IFR to estimate the numbers of COVID-19 deaths.

The key observation here is that the estimated national COVID-19 death counts offered by these studies are *lower* than the all-cause mortality estimated by Jha et al. [5], Huang et al. [4], and Xiao et al. [8] (Table 2), by about a million or more; and they were *lower* than the estimate of 4.96 million all-cause deaths when we performed age standardization and applied the age-specific mortality ratio of Zhang et al. (2024) [2] to the entire mainland China (Supplementary Table S6).

To estimate infection count, three models explicitly simulated the transmission dynamics of COVID-19 using different model designs: the individual-based model in Cheng et al. [18], the stochastic compartmental model in Cai et al. [26], and the compartmental metapopulation model in Leung et al. [28]. The other three models did not simulate transmission dynamics. Amemiya et al. used final-size equations derived from compartmental models and, therefore, implicitly depended on the assumptions of compartmental models [17]. In the individual-based model of Du et al., the extent of the COVID-19 wave was taken from empirical data from China CDC’s sentinel surveillance [19]. Ioannidis et al. made simple assumptions on the extent of the pandemic wave to calculate mortality estimates [20].

The IFR is key to estimating the COVID-19 death count from the infection count. Our Supplementary Tables S11, S12, S16, and S17 summarize the age-stratified IFR used in these studies. One potential source of differences in results between the studies was how the elderly population was stratified by age group. Some studies grouped the elderly into more age groups than other studies. Given that the risk of COVID-19 death is highly age-dependent and that older senior citizens, if infected, are more likely to die than younger senior citizens, the age structure of the models and, therefore, the IFR parameters they used might have a profound impact on the simulation results.

# Supplementary Text S5

## Importance in the history of public health

The end of the Zero COVID policy, followed by a massive COVID-19 wave in mainland China, was monumental in public health history. Never before in human history has one-fifth of the world’s population been placed under a stringent regime of public health and social measures, enforced for three consecutive years at great socioeconomic costs against a pandemic of a novel respiratory infection. Mainland China essentially postponed its pandemic wave for three years. In the ensuing three years, a majority of mainland China’s population was vaccinated with inactivated vaccines, which lowered but did not eliminate the risk of hospitalization and mortality [30]. However, the inactivated vaccine failed to significantly reduce susceptibility to infection with the Omicron variant [21]. Then, mainland China ended its Zero COVID policy on 7 December 2022. According to Du et al. [19], China CDC’s sentinel surveillance found that about nine in ten people in mainland China were infected from 16 December 2022 through 19 January 2023. This synchronization of a COVID-19 wave of over 1.4 billion people through the implementation and subsequent sudden termination of public health and social measures was an unprecedented public health “experiment.” Even though most Chinese citizens were vaccinated with inactivated vaccines [36], immunity wanes without boosters. Mainland China missed the opportunity to vaccinate its citizens with updated mRNA vaccines that were proven effective against the Omicron variant [30]. Scenario analyses made by modelers (Supplementary Texts S2-S3) showed that there were pathways that mainland China could have ended its Zero COVID policy in a scheduled manner, so that healthcare facilities could be less overwhelmed and some deaths could have been prevented [17; 26; 28]. In fact, Shanghai-based scientists made such recommendations half a year before the policy’s termination [26].

Furthermore, mainland China’s definition of COVID-19 deaths was different from that of the rest of the world [37; 38]. This made the international comparison of COVID-19 mortality data difficult. Globally, as per the World Health Organization, COVID-19 death is “defined for surveillance purposes as a death resulting from a clinically compatible illness in a probable or confirmed COVID-19 case, unless there is a clear alternative cause of death that cannot be related to COVID-19 disease (e.g., trauma). There should be no period of complete recovery between the illness and death” [39]. In contrast, mainland China adopted a narrow definition of COVID-19 deaths as “SARS-CoV-2 PCR test positive, SARS-CoV-2 induced respiratory failure directly led to deaths” as discussed by Zhang et al. [3]. While underreporting and underestimating cases and infections are global phenomena, adopting a different COVID-19 death definition distorts the global picture of the pandemic. The COVID-19 wave in December 2022–January 2023 in mainland China made this unique feature so apparent that future students of the history of epidemiology should take note. While some commentators argued that being conservative in counting disease cases has been China’s routine practice [40], skeptics might wonder if using an alternative COVID-19 death definition was part of China’s strategy to appear better than other countries’ performance during the pandemic [41].

# References

[1] **Fang X** (2024) Lùnwén pù fèichú qīng líng zhèngcè hòu shànghǎi yī qū chāo èr wàn rén sǐwáng [Research articles expose over 20,000 people died in a district in Shanghai after Zero COVID was terminated] [in Chinese] (Jan 23, 2024). Available at https://www.epochtimes.com/gb/24/1/23/n14164724.htm (accessed July 8, 2024).

[2] **Zhang M, Zhou D and Yan W** (2024) Shànghǎi mǒu qū xīnxíng guānzhuàng bìngdú liúxíng qíjiān mànxìngbìng chāo'é sǐwáng qíngkuàng diàochá [A study of excess mortality from chronic diseases during the COVID-19 pandemic in a district of Shanghai] [in Chinese]. *Shanghai Yu Fang Yi Xue [Shanghai Journal of Preventive Medicine]*.

[3] **Zhang M, et al.** (2023) Excess death and years of life lost of the residents in a town of Shanghai after the control of COVID-19 liberalized [in Chinese]. *Shanghai Yu Fang Yi Xue [Shanghai Journal of Preventive Medicine]* **35**(10)**,** 1039-1043.

[4] **Huang L, Li OZ and Yin X** (2023) Inferring China's excess mortality during the COVID-19 pandemic using online mourning and funeral search volume. *Scientific Reports* **13**(1)**,** 15665. https://doi.org/10.1038/s41598-023-42979-1.

[5] **Jha P, et al.** (2024) Excess deaths in China during SARS-CoV-2 viral waves in 2022-2023. *Preventive Medicine Reports* **41,** 102687. https://doi.org/10.1016/j.pmedr.2024.102687.

[6] **Raphson L and Lipsitch M** (2024) Estimated Excess Deaths Due to COVID-19 Among the Urban Population of Mainland China, December 2022 to January 2023. *Epidemiology* **35**(3)**,** 372-376. https://doi.org/10.1097/ede.0000000000001723.

[7] **Wang JL, et al.** (2024) Forecast of peak infection and estimate of excess deaths in COVID-19 transmission and prevalence in Taiyuan City, 2022 to 2023. *Infectious Disease Modelling* **9**(1)**,** 56-69. https://doi.org/10.1016/j.idm.2023.11.005.

[8] **Xiao H, et al.** (2023) Excess All-Cause Mortality in China After Ending the Zero COVID Policy. *JAMA Network Open* **6**(8)**,** e2330877. https://doi.org/10.1001/jamanetworkopen.2023.30877.

[9] **Wells GA, et al.** (2021) The Newcastle-Ottawa Scale (NOS) for assessing the quality of nonrandomised studies in meta-analyses. Available at https://www.ohri.ca/programs/clinical_epidemiology/oxford.asp (accessed September 28, 2024).

[10] **Mata DA, et al.** (2015) Prevalence of Depression and Depressive Symptoms Among Resident Physicians: A Systematic Review and Meta-analysis. *JAMA* **314**(22)**,** 2373-2383. https://doi.org/10.1001/jama.2015.15845.

[11] **Guójiā tǒngjì jú rénkǒu hé jiùyè tǒngjì sī [Population and Employment Statistics Division of the National Bureau of Statistics of China]** (2021) *China Population and Employment Statistics Yearbook—2021 [in Chinese]*. China Statistics Press Company Limited.

[12] **Guówùyuàn dì qī cì quánguó rénkǒu pǔchá lǐngdǎo xiǎozǔ bàngōngshì [Office of the Leading Group for the Seventh National Population Census of the State Council of China]** (2022) *China's Population Census by County - 2020 [in Chinese]*. China Statistics Press Company Limited.

[13] **National Bureau of Statistics of China** (2021) Communiqué of the Seventh National Population Census (No. 3) (2021-05-11). Available at https://www.stats.gov.cn/english/PressRelease/202105/t20210510_1817188.html (accessed July 9, 2024).

[14] **Shànghǎi shì tǒngjì jú [Shanghai Municipal Bureau of Statistics] and Guójiā tǒngjì jú shànghǎi diàochá zǒngduì [Shanghai Survey Office of the National Bureau of Statistics of China]** (2024) Statistical Bulletin of Shanghai Municipality on National Economic and Social Development in 2023 [in Chinese]. Available at https://tjj.sh.gov.cn/tjgb/20240321/f66c5b25ce604a1f9af755941d5f454a.html (accessed July 29, 2024).

[15] **Statistica** (2024) Market share of leading search engines in China from June 2019 to June 2024. Available at https://www.statista.com/statistics/253340/market-share-of-search-engines-in-china-pageviews/ (accessed July 29, 2024).

[16] **Ioannidis JPA** (2024) Analyses of academician cohorts generate biased pandemic excess death estimates. *Journal of Clinical Epidemiology* **173,** 111437. https://doi.org/10.1016/j.jclinepi.2024.111437.

[17] **Amemiya Y, Li T and Nishiura H** (2023) Age-dependent final size equation to anticipate mortality impact of COVID-19 in China. *Mathematical Biosciences and Engineering : MBE* **20**(6)**,** 11353-11366. https://doi.org/10.3934/mbe.2023503.

[18] **Cheng Q, et al.** (2023) Feasible intervention combinations for achieving a safe exit of the Zero-COVID policy in China and its determinants: an individual-based model study. *BMC Infectious Diseases* **23**(1)**,** 390. https://doi.org/10.1186/s12879-023-08382-x.

[19] **Du Z, et al.** (2023) Estimate of COVID-19 Deaths, China, December 2022-February 2023. *Emerging Infectious Diseases* **29**(10)**,** 2121-2124. https://doi.org/10.3201/eid2910.230585.

[20] **Ioannidis JPA, Zonta F and Levitt M** (2023) Estimates of COVID-19 deaths in Mainland China after abandoning zero COVID policy. *European Journal of Clinical Investigation* **53**(4)**,** e13956. https://doi.org/10.1111/eci.13956.

[21] **Suah JL, et al.** (2022) Real-world effectiveness of homologous and heterologous BNT162b2, CoronaVac, and AZD1222 booster vaccination against Delta and Omicron SARS-CoV-2 infection. *Emerging Microbes & Infections* **11**(1)**,** 1343-1345. https://doi.org/10.1080/22221751.2022.2072773.

[22] **Jara A, et al.** (2021) Effectiveness of an Inactivated SARS-CoV-2 Vaccine in Chile. *New England Journal of Medicine* **385**(10)**,** 875-884. https://doi.org/10.1056/NEJMoa2107715.

[23] **Jara A, et al.** (2022) Effectiveness of homologous and heterologous booster doses for an inactivated SARS-CoV-2 vaccine: a large-scale prospective cohort study. *Lancet Global Health* **10**(6)**,** e798-e806. https://doi.org/10.1016/S2214-109X(22)00112-7.

[24] **Sasanami M, Kayano T and Nishiura H** (2022) Monitoring the COVID-19 immune landscape in Japan. *International Journal of Infectious Diseases* **122,** 300-306. https://doi.org/10.1016/j.ijid.2022.06.005.

[25] **O'Driscoll M, et al.** (2021) Age-specific mortality and immunity patterns of SARS-CoV-2. *Nature* **590**(7844)**,** 140-145. https://doi.org/10.1038/s41586-020-2918-0.

[26] **Cai J, et al.** (2022) Modeling transmission of SARS-CoV-2 Omicron in China. *Nature Medicine* **28**(7)**,** 1468-1475. https://doi.org/10.1038/s41591-022-01855-7.

[27] **Zhang J, et al.** (2019) Patterns of human social contact and contact with animals in Shanghai, China. *Scientifc Reports* **9**(1)**,** 15141. https://doi.org/10.1038/s41598-019-51609-8.

[28] **Leung K, Leung GM and Wu JT** (2022) Modelling the adjustment of COVID-19 response and exit from dynamic zero-COVID in China. *medRxiv*. https://doi.org/10.1101/2022.12.14.22283460.

[29] **Covid-19 Forecasting Team** (2022) Variation in the COVID-19 infection-fatality ratio by age, time, and geography during the pre-vaccine era: a systematic analysis. *Lancet* **399**(10334)**,** 1469-1488. https://doi.org/10.1016/S0140-6736(21)02867-1.

[30] **McMenamin ME, et al.** (2022) Vaccine effectiveness of one, two, and three doses of BNT162b2 and CoronaVac against COVID-19 in Hong Kong: a population-based observational study. *Lancet Infectious Diseases* **22**(10)**,** 1435-1443. https://doi.org/10.1016/S1473-3099(22)00345-0.

[31] **Gan N** (2022) Shanghai is finally ‘reopening,’ but the trauma of lockdown lives on (Jun 1, 2022). Available at https://www.cnn.com/2022/06/01/china/shanghai-lockdown-reopening-intl-hnk-mic/index.html (accessed July 8, 2024).

[32] **Mistry D, et al.** (2021) Inferring high-resolution human mixing patterns for disease modeling. *Nature Communications* **12**(1)**,** 323. https://doi.org/10.1038/s41467-020-20544-y.

[33] **Guan WJ, et al.** (2020) Clinical Characteristics of Coronavirus Disease 2019 in China. *New England Journal of Medicine* **382**(18)**,** 1708-1720. https://doi.org/10.1056/NEJMoa2002032.

[34] **Leung K, Wu JT and Leung GM** (2021) Effects of adjusting public health, travel, and social measures during the roll-out of COVID-19 vaccination: a modelling study. *Lancet Public Health* **6**(9)**,** e674-e682. https://doi.org/10.1016/S2468-2667(21)00167-5.

[35] **Guójiā tǒngjì jú rénkǒu hé jiùyè tǒngjì sī [Population and Employment Statistics Division of the National Bureau of Statistics of China]** (2011) *China Population and Employment Statistics Yearbook—2011 [in Chinese]*. China Statistics Press Company Limited.

[36] **Wee S-L and Chen E** (2021) China says it has fully vaccinated 1 billion people (Sep 16, 2021). Available at https://www.nytimes.com/2021/09/16/world/china-covid-vaccine.html (accessed July 8, 2024).

[37] **Armstrong K** (2023) China Covid: WHO warns about under-representing Covid deaths (4 January 2023). Available at https://www.bbc.com/news/world-asia-china-64167052 (accessed October 27, 2024).

[38] **Mackintosh T** (2022) China Covid: Five deaths under country's new counting method (20 December 2022). Available at https://www.bbc.com/news/world-asia-64044204 (accessed October 27, 2024).

[39] **World Health Organization** (2022) Public health surveillance for COVID-19: interim guidance. Available at https://www.who.int/publications/i/item/who-2019-nCoV-surveillanceguidance-2020.7 (accessed September 2, 2024).

[40] **National Public Radio** (2022) China limits how it defines COVID deaths in official count (December 21, 2022). Available at https://www.npr.org/2022/12/21/1144631144/china-limits-how-it-defines-covid-deaths-in-official-count (accessed July 29, 2024).

[41] **Fu K-W** (2023) Propagandization of Relative Gratification: How Chinese State Media Portray the International Pandemic. *Political Communication* **40**(6)**,** 788-809. https://doi.org/10.1080/10584609.2023.2207492.

[42] **Chen B, et al.** (2023) Why were some countries more successful than others in curbing early COVID-19 mortality impact? A cross-country configurational analysis. *PLoS One* **18**(3)**,** e0282617. https://doi.org/10.1371/journal.pone.0282617.

[43] **Chen SX, et al.** (2023) Social media trust predicts lower COVID-19 vaccination rates and higher excess mortality over 2 years. *PNAS Nexus* **2**(10)**,** pgad318. https://doi.org/10.1093/pnasnexus/pgad318.

[44] **Du P, et al.** (2023) A continuous age-specific standardized mortality ratio for estimating the unascertained rates in the early epidemic of COVID-19 in different regions. *Journal of Applied Statistics* **50**(11-12)**,** 2504-2517. https://doi.org/10.1080/02664763.2021.1947995.

[45] **Ioannidis JPA, Zonta F and Levitt M** (2023) Variability in excess deaths across countries with different vulnerability during 2020-2023. *Proceedings of the National Academy of Sciences of the United States of America* **120**(49)**,** e2309557120. https://doi.org/10.1073/pnas.2309557120.

[46] **Musa SS, et al.** (2024) Evaluating the spike in the symptomatic proportion of SARS-CoV-2 in China in 2022 with variolation effects: a modeling analysis. *Infectious Disease Modelling* **9**(2)**,** 601-617. https://doi.org/10.1016/j.idm.2024.02.011.

[47] **Normile D** (2023) China is flying blind as the pandemic rages. *Science* **379**(6627)**,** 11-12. https://doi.org/10.1126/science.adg5286.

[48] **Peng S, et al.** (2024) COVID-19 risk prediction scores for mortality: A validation study from the National Registry of COVID-19 in China. *Chinese Medical Journal (English)* **137**(6)**,** 743-745. https://doi.org/10.1097/cm9.0000000000002973.

[49] **Xu X, et al.** (2024) Impact of COVID-19 on risks and deaths of non-communicable diseases in the Western Pacific region. *Lancet Regional Health - Western Pacific* **43,** 100795. https://doi.org/10.1016/j.lanwpc.2023.100795.

[50] **Zou F, et al.** (2024) Multilayer factors associated with excess all-cause mortality during the omicron and non-omicron waves of the COVID-19 pandemic: time series analysis in 29 countries. *BMC Public Health* **24**(1)**,** 350. https://doi.org/10.1186/s12889-024-17803-8.

[51] **Dang O, Kamiya S and Zhou L** (2025) The Impact of COVID-19 on Mortality in 34 Countries and Economies. *North American Actuarial Journal***,** 1-38. https://doi.org/10.1080/10920277.2025.2496726.

[52] **Shang W and Liu M** (2023) Summary of COVID-19 surveillance systems and analysis on surveillance data in the world, taking the WHO, Europe, United Kingdom, United States, Japan and China for example [in Chinese]. *Zhonghua Liu Xing Bing Xue Za Zhi [Chinese Journal of Epidemiology]* **44**(2)**,** 190-195.

[53] **Zhu Q, et al.** (2024) Survey on COVID-19 among residents in Anhui province in the new stage of epidemic prevention and control [in Chinese]. *Anhui Yi Ke Da Xue Xue Bao [Acta Universitatis Medicinalis Anhui]* **59**(8)**,** 1455-1459.

[54] **Shao W, et al.** (2022) Effectiveness of COVID-19 vaccines against SARS-CoV-2 variants of concern in real-world: a literature review and meta-analysis. *Emerging Microbes & Infections* **11**(1)**,** 2383-2392. https://doi.org/10.1080/22221751.2022.2122582.

[55] **Yan VKC, et al.** (2022) Effectiveness of BNT162b2 and CoronaVac vaccinations against mortality and severe complications after SARS-CoV-2 Omicron BA.2 infection: a case-control study. *Emerging Microbes & Infections* **11**(1)**,** 2304-2314. https://doi.org/10.1080/22221751.2022.2114854.

[56] **Yang B, et al.** (2022) Effectiveness of CoronaVac and BNT162b2 Vaccines Against Severe Acute Respiratory Syndrome Coronavirus 2 Omicron BA.2 Infections in Hong Kong. *Journal of Infectious Diseases* **226**(8)**,** 1382-1384. https://doi.org/10.1093/infdis/jiac360.

[57] **Wang XY, et al.** (2022) Efficacy of heterologous boosting against SARS-CoV-2 using a recombinant interferon-armed fusion protein vaccine (V-01): a randomized, double-blind and placebo-controlled phase III trial. *Emerging Microbes & Infections* **11**(1)**,** 1910-1919. https://doi.org/10.1080/22221751.2022.2088406.

[58] **Davies NG, et al.** (2021) Increased mortality in community-tested cases of SARS-CoV-2 lineage B.1.1.7. *Nature* **593**(7858)**,** 270-274. https://doi.org/10.1038/s41586-021-03426-1.

[59] **Twohig KA, et al.** (2022) Hospital admission and emergency care attendance risk for SARS-CoV-2 delta (B.1.617.2) compared with alpha (B.1.1.7) variants of concern: a cohort study. *Lancet Infectious Diseases* **22**(1)**,** 35-42. https://doi.org/10.1016/S1473-3099(21)00475-8.

# Supplementary Tables

**Supplementary Table S1.** Inclusion and exclusion criteria used in this systematic review of excess mortality in mainland China due to the pandemic wave after the end of the Zero COVID policy on 7 December 2022.

| Category | Inclusion | Exclusion |
| --- | --- | --- |
| Study location | Mainland China | Not mainland China (e.g., Hong Kong) |
| Study period | The COVID-19 wave that followed the end of Zero COVID policy (2022-12-07)  Operationally defined as:  From 2022-12-07 To 2023-03-31 | Did not include the time period of the COVID-19 wave that followed the end of the Zero COVID policy (2022-12-07) |
| Excess mortality | Must include estimation of excess deaths (excess mortality) due to the end of the Zero COVID policy at the population level | Did not include an estimation of excess deaths (excess mortality) due to the end of the Zero COVID policy at the population level |
| Language | English or Chinese | Other languages |
| Genre | Research articles (including brief reports) published in peer-reviewed journals | Reviews, commentaries, editorials, and letters to the editors that did not include peer-reviewed original research.  Grey literature (i.e., not peer-reviewed journals).  Preprint only (e.g., medRxiv). |

**Supplementary Table S2.** Reasons for exclusion after full-text review.

| Article | Reasons | Detailed explanations |
| --- | --- | --- |
| **English language article** |  |  |
| Amemiya Y et al. (2023) Age-dependent final size equation to anticipate mortality impact of COVID-19 in China. Mathematical Biosciences and Engineering. 20(6):11353-11366. [17] | No estimates of excess deaths due to the end of the Zero COVID policy | Estimated mortality but not excess mortality due to the end of the Zero COVID policy  (Included in Supplementary Text S2) |
| Chen B et al. (2023) Why were some countries more successful than others in curbing early COVID-19  mortality impact? A cross-country configurational analysis. PLoS ONE. 18(3):e0282617. [42] | Did not include the time period that followed the end of the Zero COVID-19 policy | Used 6 January 2021 as data cut-off point. |
| Chen SX et al. (2023) Social media trust predicts lower COVID-19 vaccination rates and higher excess mortality over 2 years. PNAS Nexus. 2(10):pgad318. [43] | Did not include the time period that followed the end of the Zero COVID-19 policy  No estimates of excess deaths due to the end of the Zero COVID policy | Used study points of 1 April 2021, 24 November 2021, and 1 April 2022.  Did not estimate excess mortality but took excess mortality data from other sources. |
| Cheng Q et al. (2023) Feasible intervention combinations for achieving a safe exit of the Zero-COVID policy in China and its determinants: an individual-based model study. BMC Infectious Diseases. 23:390. [18] | No estimates of excess deaths due to the end of the Zero COVID policy | Estimated mortality but not excess mortality due to the end of the Zero COVID policy  (Included in Supplementary Text S2) |
| Du P et al. (2023) A continuous age-specific standardized mortality ratio for estimating the unascertained rates in the early epidemic of COVID-19 in different regions. Journal of Applied Statistics. 50(11-12):2504-2517. [44] | Did not include the time period that followed the end of the Zero COVID-19 policy  No estimates of excess deaths due to the end of the Zero COVID policy | Used confirmed case and death count data from 11 January 2020 to 15 June 2020.  Estimated deaths due to COVID-19 only, did not estimate total excess deaths. |
| Du Z et al. (2023) Estimate of COVID-19 Deaths, China, December 2022-February 2023. Emerging Infectious Diseases. 29(10):2121-2124. [19] | No estimates of excess deaths due to the end of the Zero COVID policy | Estimated mortality but not excess mortality due to the end of the Zero COVID policy (Included in Supplementary Text S2) |
| Ioannidis JPA et al. (2023) Variability in excess deaths across countries with different  vulnerability during 2020–2023. Proceedings of the National Academy of Sciences of the United States of America. 120(49):e2309557120. [45] | Not mainland China  No estimates of excess deaths due to the end of the Zero COVID policy | China was not one of the 34 countries analyzed in this article.  The end of the Zero COVID-19 policy was not the focus of estimated excess deaths due to COVID-19. |
| Ioannidis JPA et al. (2023). Estimates of COVID-19 deaths in Mainland China after abandoning zero COVID policy. European Journal of Clinical Investigation. 53:e13956. [20] | No estimates of excess deaths due to the end of the Zero COVID policy | Estimated mortality but not excess mortality due to the end of the Zero COVID policy (Included in Supplementary Text S2) |
| Musa SS et al. (2024) Evaluating the spike in the symptomatic proportion of SARS-CoV-2 in China in 2022 with variolation effects: a modeling analysis. Infectious Disease Modelling. 9:601-617. [46] | No estimates of excess deaths due to the end of the Zero COVID policy | Fit compartmental model simulations to data from the WHO dashboard for COVID-19 situation reports: reported daily and cumulative case count in China. Did not estimate excess mortality from observed data. |
| Normile D. (2023) China is flying blind as the pandemic rages. Science. 379(6627).11-12. [47] | Not peer-reviewed original research  No estimates of excess deaths due to the end of the Zero COVID policy | This was a news article and not a peer-reviewed article.  Mentioned reporting of all fatalities in which COVID-19 was a factor but did not estimate excess deaths. |
| Peng S et al. (2024) COVID-19 risk prediction scores for mortality: A validation study from the National Registry of COVID-19 in China. Chinese Medical Journal (English). 137(6):743-745. [48] | Did not include the time period that followed the end of the Zero COVID-19 policy  No estimates of excess deaths due to the end of the Zero COVID policy | Used data from 1 January 2020 to 28 March 2020.  Used clinical data from COVID-19 patients to develop models of factors that predict individual patient’s COVID-19 mortality but did not estimate population-level excess mortality. |
| Xu X et al. (2024) Impact of COVID-19 on risks and deaths of non-communicable diseases in the Western Pacific region. The Lancet Regional Health – Western Pacific. 43:100795. [49] | Not peer-reviewed original research (Wrong genre, grey literature, preprint only)  No estimates of excess deaths due to the end of the Zero COVID policy | This was a narrative review article.  The article only briefly mentioned the Zero COVID-19 policy and did not estimate excess mortality. |
| Zou F et al. (2024) Multilayer factors associated with excess all-cause mortality during the omicron and non-omicron waves of the COVID-19 pandemic: time series analysis in 29 countries. BMC Public Health. 24:350. [50] | Did not include the time period that followed the end of the Zero COVID-19 policy  Not mainland China  No estimates of excess deaths due to the end of the Zero COVID policy | Used data up to the 30th week of 2022 (July 24th-30th).  Examined data from 29 countries, which did not include China.  Did not mention or estimate excess deaths due to the end of the Zero COVID policy. |
| Dang et al. (2025) The Impact of COVID-19 on Mortality in 34 Countries and Economies. North American Actuarial Journal. DOI: 10.1080/10920277.2025.2496726 [51] | Not mainland China | The scope of the study did not include mainland China |
| Ioannidis (2024) Analyses of academician cohorts generate biased pandemic excess death estimates. Journal of Clinical Epidemiology. 173:111437. [16] | No estimates of excess deaths due to the end of the Zero COVID policy | Did not estimate excess deaths due to the end of the Zero COVID policy at the population level |
| **Chinese language article** |  |  |
| Shang W and Liu M (2023). Summary of COVID-19 surveillance systems and analysis on surveillance data in the world, taking the WHO, Europe, United Kingdom, United States, Japan and China for example. Zhonghua Liu Xing Bing Xue Za Zhi [Chinese Journal of Epidemiology]. 44(2):190-195. [in Chinese] [52] | Not peer-reviewed original research  Did not include the time period that followed the end of the Zero COVID policy  No estimates of excess deaths due to the end of the Zero COVID policy | This was a narrative review article.  Only mentioned China’s cumulative reported case count and cumulative death count data as of 29 November 2022.  Did not mention or estimate excess deaths due to the end of the Zero COVID policy. |
| Zhu Q et al. (2024). Survey on COVID-19 among residents in Anhui province in the new stage of epidemic prevention and control. Anhui Yi Ke Da Xue Xue Bao [Acta Universitatis Medicinalis Anhui]. 59(8):1455-1459. [in Chinese] [53] | Unrelated to the topic  No estimates of excess deaths due to the end of the Zero COVID policy | A survey of self-reported suspect COVID-19 infection  Did not estimate excess deaths due to the end of the Zero COVID policy |

Note: Amemiya et al. [17], Cheng et al. [18], Du et al. [19], and Ioannidis et al. [20] are excluded from the main review. They are included in Supplementary Text S2.

**Supplementary Table S3.** Modified Newcastle-Ottawa Scale. The scores on the scale may range from 0 to 10. The scale assesses the risk of bias in the included studies in the following domains: sample representativeness (2) and size (2), comparability between respondents and non-respondents (2), ascertainment of the outcome of interest (2), and statistical quality (2). Studies with 6+ points were deemed to be of low risk of bias; otherwise, high risk of bias. This modified scale was adapted from the Modified Newcastle-Ottawa Scale for prevalence studies, as presented in Mata et al. [10].

| Categories and points | Explanation |
| --- | --- |
| **Representativeness of the sample** |  |
| 2 points | Official death records of the whole jurisdiction. |
| 1 point | Established the sample’s representativeness of the population. |
| 0 points | Unable to establish the sample’s representativeness of the population. |
| *Notes* | Data may not necessarily represent the population at risk, e.g., low response rate in the survey; or internet search data may not represent the entire population due to poor access to the internet by some population subgroups; or obituary data of academic institutions may not truly represent the population subgroup (e.g., age and geography) that they meant to represent; or, fail to provide a source of the death count data. Alternatively, the researchers can establish the sample’s representativeness of the population by presenting their statistical methods that do so. For example, they may establish that the trends of internet search volumes of certain keywords over time have a strong statistical correlation with mortality trends over time. |
| **Sample size** |  |
| 2 points | Official death records of the whole jurisdiction. |
| 1 point | Sample size ≥200 participants (or subpopulation from whom obituaries could be obtained, if deaths occurred). |
| 0 points | Sample size <200 participants (or source of death records is unknown). |
| **Non-respondents** |  |
| 2 points | Official death records of the whole jurisdiction. |
| 1 point | Comparability between respondents’ and non-respondents’ characteristics was established, the response rate was satisfactory; alternative: comparability between people with whom the researchers have data and those who do not. |
| 0 points | The response rate was unsatisfactory, the comparability between respondents and non-respondents was unsatisfactory, or there was no description of the response rate or the characteristics of the responders and the non-responders. |
| **Ascertainment of outcome of interest** |  |
| **(a) Estimates of the total number of deaths** |  |
| 1 point | Official death records. |
| 0.5 points | Estimates via indirect methods (including reports of family deaths by survey participants). |
| 0 point | Data provided without any source or any method of estimation described or did not estimate the total number of deaths. |
| **(b) Estimates of expected deaths (or excess deaths, or excess mortality rate, if there is no estimation of expected deaths)** |  |
| 1 point | Statistical methods were clearly described, with age and sex/gender differences in COVID-19 risks taken into consideration (e.g., stratification). |
| 0.5 points | Statistical methods were clearly described without taking age and sex/gender differences in COVID-19 risks into consideration (e.g., stratification). |
| 0 point | Statistical methods were not clearly described. |
| **Quality of descriptive and analytical statistics reporting** |  |
| 2 points | Reported descriptive statistics to describe the population (e.g., age, sex) or proxy dataset with proper measures of dispersion (e.g., standard deviation, standard error, range) and appropriate use of statistical analysis. |
| 1 point | Reported descriptive statistics to describe the population (e.g., age, sex) or proxy dataset with proper measures of dispersion (e.g., standard deviation, standard error, range), and there was a deficiency in the use of statistical analysis. |
| 0 point | Descriptive statistics were not reported, were incomplete, or did not include proper measures of dispersion. |

**Supplementary Table S4.** Risk of bias assessment results

|  | Represen-tativeness | Sample size | Non-respondents | Ascertainment of outcome of interest (total deaths) | Ascertainment of outcome of interest (expected deaths or excess deaths) | Quality of descriptive and analytical statistics reporting | TOTAL |
| --- | --- | --- | --- | --- | --- | --- | --- |
| Zhang (2023) [3] | 2 | 2 | 2 | 1 | 1 | 2 | 10 |
| Zhang (2024) [2] | 2 | 2 | 2 | 1 | 1 | 2 | 10 |
| Jha (2024) [5] | 1 | 1 | 1 | 0 | 0.5 | 2 | 5.5 |
| Huang (2023) [4] | 1 | NA | 0 | 0.5 | 0.5 | 2 | 4 |
| Raphson and Lipsitch (2024) [6] | 0 | 0 | 0 | 0 | 1 | 2 | 3 |
| Xiao (2023) [8] | 1 | 1 | 0 | 0.5 | 0.5 | 2 | 5 |
| Wang (2024) [7] | 0 | 0 | 0 | 0 | 0.5 | 1 | 1.5 |

NA: Not applied

**Supplementary Table S5.** Expected, actual, and excess mortality data as reported in Zhang et al. (2024) [2]. The excess mortality rate was recalculated as (excess deaths / expected deaths) × 100%, based on the death counts provided in the original paper.

|  | **Expected number of deaths** | | | |  |  |  |
| --- | --- | --- | --- | --- | --- | --- | --- |
|  | **7–31 Dec 2022,**  ***n* (95% CI)** | **Jan 2023,**  ***n* (95% CI)** | **1–23 Feb 2023,**  ***n* (95% CI)** | **Total** | **Actual number of deaths** | **Excess number of deaths** | **Excess mortality rate (%)** |
| Sex |  |  |  |  |  |  |  |
| Male | 1659.53  (1383.07-1936.00) | 1700.70  (1378.29-2023.12) | 1504.37  (1141.78-1866.96) | 4274.76 | 12257 | 7982.24 | 186.73 |
| Female | 1287.88  (1026.56-1549.20) | 1337.05  (1018.07-1656.04) | 1236.72  (869.00-1604.44) | 3391.54 | 8733 | 5341.46 | 157.49 |
| Age group |  |  |  |  |  |  |  |
| ≤44 years | 107.94  (93.98-121.90) | 110.38  (96.36-124.41) | 91.38  (77.30-105.47) | 272.49 | 342 | 69.51 | 25.51 |
| 45-59 years | 199.03  (164.26-233.80) | 192.19  (153.32-231.07) | 179.86  (137.28-222.45) | 500.44 | 699 | 198.56 | 39.68 |
| 60-74 years | 758.15  (624.76-891.54) | 766.65  (611.10-922.20) | 670.98  (496.05-845.92) | 1929.22 | 4193 | 2263.78 | 117.34 |
| 75-89 years | 1248.14  (1002.12-1494.15) | 1344.80  (1029.75-1659.86) | 1211.47  (840.00-1582.94) | 3346.50 | 10180 | 6833.50 | 204.20 |
| ≥90 years | 638.07  (498.40-777.73) | 630.57  (467.69-793.44) | 594.23  (411.07-777.40) | 1633.26 | 5576 | 3942.74 | 241.40 |
| Chronic disease |  |  |  |  |  |  |  |
| Hypertension | 59.91  (41.54-78.28) | 68.49  (44.97-92.01) | 64.82  (37.1-92.55) | 170.05 | 636 | 465.95 | 274.01 |
| Diabetes | 48.55  (20.17-76.94) | 47.39  (12.74-82.04) | 42.72  (2.78-82.66) | 122 | 570 | 448.37 | 367.52 |
| Tumor | 485.69  (430.63-540.75) | 485.86  (411.78-559.94) | 425.53  (336.4-514.65) | 1227 | 1566 | 338.91 | 27.62 |
| Coronary heart disease | 181.43  (124.91-237.94) | 171.43  (105.53-237.33) | 165.43  (91.31-239.54) | 454 | 1419 | 965.37 | 212.64 |
| Cerebral infarction | 173.65  (109.36-237.94) | 170.49  (88.15-252.82) | 147.49  (50.41-244.56) | 431.68 | 1394 | 962.32 | 222.92 |
| Pulmonary infection | 182.12  (130.16-234.08) | 218.29  (166.07-270.5) | 202.96  (150.48-255.43) | 532 | 2312 | 1780.12 | 334.61 |
| Others | 1834.95  (1515.2-2154.71) | 1904.62  (1514.32-2294.92) | 1720.95  (1271.03-2170.88) | 4798 | 13093 | 8294.94 | 172.88 |
| Total | 2941.74  (2408.24-3475.24) | 3032.08  (2380.86-3683.30) | 2735.41  (1984.71-3486.11) | 7651.39 | 20990 | 13338.61 | 174.33 |

CI, confidence interval.

**Supplementary Table S6.** Estimation of the number of deaths across mainland China from 7 December 2022 to 23 February 2023, assuming the same age-stratified mortality rates as observed in Zhang et al. (2024) [2]. We assumed that (a) mortality data reported in Zhang et al. (2024) was from Pudong New District, the district with the highest population size in Shanghai, to obtain (b) conservative estimates of mortality rates and then (c) apply them to mainland China’s population size by age stratum to obtain the estimated death count.

| (a) | Number of actual deaths [2] † | Number of expected deaths [2] † | Number of excess deaths [2] † | Pudong New District population size [12] |
| --- | --- | --- | --- | --- |
| ≤44y | 342 | 272.49 | 69.51 | 3,318,361 |
| 45-59y | 699 | 500.44 | 198.56 | 1,144,243 |
| 60-74y | 4193 | 1929.22 | 2263.78 | 950,827 |
| ≥75y | 15756 | 4979.66 | 10776.24 | 278,081 |
| Total | 20990 | - | - | 5,691,512 |
| (b) | **Actual deaths as a % of population size (%)** | **Expected deaths as a % of population size (%)** | **Excess deaths as a % of population size (%)** |  |
| ≤44y | 0.0103 | 0.0082 | 0.0021 |  |
| 45-59y | 0.0611 | 0.0437 | 0.0174 |  |
| 60-74y | 0.4410 | 0.2029 | 0.2381 |  |
| ≥75y | 5.6660 | 1.7908 | 3.8752 |  |
| (c) | **Number of estimated deaths** | **Number of expected deaths** | **Number of excess deaths** | **Mainland China population size [11]** |
| ≤44y | 83,375 | 66,429.29 | 16,945.58 | 808,970,537 |
| 45-59y | 205,740 | 147,296.66 | 58,443.02 | 336,789,969 |
| 60-74y | 868,645 | 399,667.79 | 468,977.08 | 196,978,534 |
| ≥75y | 3,798,452 | 1,200,519.05 | 2,597,932.70 | 67,039,684 |
| Total | 4,956,211 | 1,813,912.79 | 3,142,298.38 | 1,409,778,724 |

y: years. †The numbers presented here for the ≥75y age groups were the summation of the numbers for both 75-89y and ≥90y age groups as presented in the paper. The number of actual deaths should be exact. However, the number of expected deaths, and therefore excess deaths, could be slightly different because the sum of the expected deaths across age groups was not equal to the expected deaths for the whole population, as reported in the paper. This had to do with the estimation method and how data was categorized by age group. We had to group 75-89y and ≥90y together because the highest age group in the district-level demographic data was ≥85y.

**Supplementary Table S7.** Chinese terms and their English translation used in the Baidu Index analysis performed by Huang et al. [4] and Xiao et al. [8]. The Mandarin transliteration of Chinese terms is provided according to the phonetic system *pinyin*.

| English translation | Terms in simplified Chinese characters | Mandarin transliteration |
| --- | --- | --- |
| **Huang et al. [4]** |  |  |
| Wreath and elegiac couplet | 花圈挽联 | Huāquān wǎnlián |
| Obituary | 讣告 | Fùgào |
| Mortuary house | 殡仪馆 | Bìnyíguǎn |
| Cinerary Casket | 骨灰盒 | Gǔhuī hé |
| Cremation | 火化 | Huǒhuà |
| Pass away | 逝世 | Shìshì |
| **Xiao et al. [8]** |  |  |
| Funeral parlor | 殡仪馆 | Bìnyíguǎn |
| Cremation | ⽕葬 | Huǒ zàng |
| Crematorium | ⽕葬场 | Huǒ zàng chǎng |
| Burial | ⼟葬 | Tǔ zàng |

**Supplementary Table S8.** Summary of methods and data used by the four papers reviewed in Supplementary Text S2.

|  | Methods | Data |
| --- | --- | --- |
| Amemiya et al. [17] | Age-structured SIR model-derived final size equation, accounting for vaccination-reduced susceptibility | VE from ref. [21-23; 54-56]. Contact matrix from Japan [24]. IFR data from O’Driscoll et al. [25]. |
| Cheng et al. [18] | Individual-based transmission model of 500,000 people. Interventions analyzed included increasing facial covering, increased healthcare capacity, vaccine coverage, and antiviral treatment coverage. | VE from ref. [26; 54]. Contact matrix data (all and only home settings) from Shanghai in 2017 [27]. |
| Du et al. [19] | Individual-based model of 1 million people estimating daily death count from case count from sentinel surveillance, accounting for VE and vaccine coverage. This is not a transmission model. | China CDC sentinel surveillance’s daily test positivity reports. IFR data from Leung, Leung and Wu [28]. Reported daily age-specific vaccination rates from Cai et al. [26]. |
| Ioannidis et al. [20] | Estimated the age-specific IFRs using data from Hong Kong and South Korea and then applied them to the population of mainland China. In the worst-case scenario, they assumed the entire population there would be infected. | Data from Hong Kong and South Korea (population size, COVID-19 deaths, and cases) by age group; Population size of mainland China by age group. |

China CDC: Chinese Center for Disease Control and Prevention. IFR: Infection-fatality ratio. SIR: Susceptible-Infectious-Recovered. VE: vaccine effectiveness.

**Supplementary Table S9.** Summary of estimated death count and selected sensitivity/scenario analysis of the four papers reviewed in Supplementary Text S2.

|  | Estimated death count | Selected sensitivity/ scenario analysis |
| --- | --- | --- |
| Amemiya et al. [17] | Assuming an R_0_ of 3.4, and assuming a second-dose VE at 0%, 10%, and 20%, respectively, there would be 1.47, 1.46, and 1.44 million deaths, respectively. | 1. Assuming a second-dose VE of 0%, 10%, and 20%, respectively: (a) a 10% random increase in third-dose coverage would prevent 30948, 24106, and 16367 deaths; (b) a 20% random increase in the third-dose coverage would prevent 64868, 49717, and 33228 deaths, respectively; (c) a 10% reduction in third-dose coverage would increase the number of deaths by 28337, 22732, and 15899 deaths, respectively.  2. Assuming an R_0_ of 3.4, if the mRNA (BNT162b2) vaccine was used instead of inactivated vaccines, the estimated death count would be 1.1 million instead. Under this scenario, if a second-dose effectiveness of 0%, 10%, and 20% was assumed, the number of deaths prevented would be 343,000, 325,000, and 306,000, respectively. |
| Cheng et al. [18] | Baseline *worst-case* scenario: 83.7 per 100,000 persons. This translated into a death count of 1,179,985.† | Baseline scenario: on *average*, 29.3 per 100,000 persons. This translated into a death count of 413,065. |
| Du et al. [19] | 1.41 million (95% CrI, 1.14-1.73 million) | Six sensitivity analyses.  S1: If the vaccine was ineffective, 5.11 million (95% CrI, 4.15-6.28 million) deaths.  S2: VE does not decline after six months: 1.32 million (1.06-1.62 million).  S3: IFR increased by 3.39-fold for 3 days at the epidemic peak: 2.11 million (1.71 – 2.60 million).  S4: model of 2 million: 1.43 million (1.16 – 1.76 million).  S5: model of 500,000: 1.43 million (1.13 – 1.76 million).  S6: Alternative age-specific VE: 1.56 million (1.28 – 1.93 million) |
| Ioannidis et al. [20] | Main scenario: only 45% of the 0-59-year-olds were infected, and the fatality impact among ≥60-year-olds was reduced by 50%: 249,094 (Hong Kong IFR) or 152,886 (South Korea IFR). | The worst-case scenario when everyone would be infected and with the same IFR as Hong Kong and South Korea: 987,455 (Hong Kong IFR) and 619,549 (South Korea IFR). Ioannidis et al. varied the fraction of the non-elderly population who would be infected and the fatality impact experienced by the elderly. |

†Assuming a population size of 1,409,778,724 in mainland China, according to China Statistical Yearbook 2021 [11], the same number used in Du et al. [19]. CrI: Credible interval. IFR: Infection-fatality ratio. R_0_: Basic reproduction number. VE: vaccine effectiveness.

**Supplementary Table S10.** Data for age-group-specific population size in mainland China used in Amemiya et al. [17], Du et al. [19] and Ioannidis et al. [20].

|  | Amemiya et al. [17] | Du et al. [19] | Ioannidis et al. [20] * |
| --- | --- | --- | --- |
| Source | World Population Prospects 2022 by United Nations | China Statistical Yearbook 2021 | Pyramidnet.net |
| Age group (years) |  |  |  |
| 0-9 | 167,379,306 | 168,127,944 | 335,323,469 |
| 10-19 | 167,944,163 | 157,940,134 |  |
| 20-29 | 175,108,854 | 166,789,007 | 175,108,854 |
| 30-39 | 233,767,164 | 223,158,122 | 233,767,164 |
| 40-49 | 202,957,964 | 207,180,217 | 202,957,964 |
| 50-59 | 233,838,693 | 222,565,082 | 233,838,693 |
| 60-69 | 155,777,903 | 147,388,498 | 154,777,903 |
| 70-79 | 82,730,213 | 80,828,885 | 81,730,213 |
| 80-89 | 28,878,906 | 35,800,835 | 23,834,546 |
| ≥90 |  |  | 3,132,598 |
| Long-term care facilities | Not applied | Not applied | 4,000,000 |
| Total | 1,448,383,166 | 1,409,778,724 | 1,448,471,404 |

*Ioannidis et al.’s [20] data was very similar to that of Amemiya et al. [17], with minor differences in the 80+ age groups. Ioannidis assumed that 1 million of 60-69 year-olds, 1 million of 70-79 year-olds, 1.5 million of 80-89 year-olds, and 0.5 million of ≥90 year-olds lived in long-term care facilities in mainland China.

**Supplementary Table S11.** Age-group-specific infection-fatality ratio (IFR) values used in Amemiya et al. [17], Du et al. [19] and Ioannidis et al. [20].

| Age group (years) | Amemiya et al. [17]* (%) | Du et al. [19]† (%) | Ioannidis et al. [20]: Hong Kong IFR (%) | Ioannidis et al. [20]: South Korea IFR (%) |
| --- | --- | --- | --- | --- |
| 0-9 | 0.002 | 0.0005 (0.0004, 0.0008) | 0.0017 | 0.0006 |
| 10-19 | 0.002 | 0.0005 (0.0003, 0.0008) |  |  |
| 20-29 | 0.0095 | 0.0005 (0.0004, 0.0008) | 0.0027 | 0.0012 |
| 30-39 | 0.032 | 0.023 (0.016, 0.034) | 0.0035 | 0.0017 |
| 40-49 | 0.098 | 0.023 (0.016, 0.036) | 0.0083 | 0.0052 |
| 50-59 | 0.265 | 0.126 (0.088, 0.196) | 0.036 | 0.0177 |
| 60-69 | 0.7655 | 0.126 (0.087, 0.198) | 0.0825 | 0.0575 |
| 70-79 | 2.4385 | 2.00 (1.38, 3.15) | 0.1757 | 0.2338 |
| 80-89 | 8.292 | 8.70 (6.12, 13.01) | 0.6506 | 0.5779 |
| ≥90 |  |  | 2.6024 | 2.3114 |
| Long-term care facilities | Not applied | Not applied | 9 | 1.7341 |

* Data taken from O’Driscoll [25]. † Du et al. [19] used the ratios of lower and upper 95% confidence intervals to the mean in the paper by the COVID-19 Forecasting Team [29] to scale the estimates in Leung, Leung, and Wu [28].

**Supplementary Table S12.** Age-group-specific transition rates used in Cheng et al. [18] if an individual was unvaccinated. They used the same method to estimate as in Cai et al. [26] but used age-specific case-fatality rates reported in Hong Kong on 12 January 2023 instead of Spring 2022.

| Age group (years) | Proportion of asymp-tomatic cases | Probability of becoming a severe case from a mild case | Probability of becoming a critical case from a mild case | Probability of death from a severe case | Probability of death from a critical case | IFR (%)* |
| --- | --- | --- | --- | --- | --- | --- |
| [0,5) | 0.973 | 0.08 | 0 | 0.004 | 0.034 | 0.00086 |
| [5,10) | ‘’ | 0.057 | 0.001 | 0.005 | 0.05 | 0.00090 |
| [10,15) | ‘’ | 0.022 | ‘’ | 0.007 | 0.067 | 0.00060 |
| [15,20) | ‘’ | ‘’ | ‘’ | ‘’ | 0.1 | 0.00069 |
| [20,25) | 0.966 | 0.036 | 0.002 | 0.01 | 0.125 | 0.00207 |
| [25,30) | ‘’ | ‘’ | ‘’ | ‘’ | ‘’ | ‘’ |
| [30,35) | ‘’ | 0.046 | 0.005 | 0.008 | 0.104 | 0.00302 |
| [35,40) | ‘’ | ‘’ | ‘’ | ‘’ | ‘’ | ‘’ |
| [40,45) | 0.954 | 0.032 | 0.006 | 0.032 | 0.207 | 0.01042 |
| [45,50) | ‘’ | ‘’ | ‘’ | ‘’ | ‘’ | ‘’ |
| [50,55) | ‘’ | 0.07 | 0.017 | 0.058 | 0.333 | 0.04472 |
| [55,60) | ‘’ | ‘’ | ‘’ | ‘’ | ‘’ | ‘’ |
| [60,65) | 0.947 | 0.079 | 0.033 | 0.087 | 0.364 | 0.10009 |
| [65,70) | ‘’ | ‘’ | ‘’ | ‘’ | ‘’ | ‘’ |
| [70,75) | ‘’ | 0.194 | 0.18 | 0.25 | 0.375 | 0.61480 |
| [75,Inf) | 0.903 | 0.251 | 0.234 | ‘’ | ‘’ | 1.45985 |

*IFR (%) is calculated as (1 - the proportion of asymptomatic cases) × (probability of becoming a severe case from a mild case × probability of death from a severe case + probability of becoming a critical case from a mild case × probability of death from a critical case). These IFR values were not provided by Cheng et al. in their paper [18]; we calculated them for this systematic review. ‘’: the same as above.

**Supplementary Table S13.** Summary of methods and data used of two papers reviewed in Supplementary Text S3.

|  | Methods | Data |
| --- | --- | --- |
| Cai et al. [26] | Age-structured stochastic compartmental model | Contact matrix data from pre-pandemic China [32]. Calibrated the transmissibility and proportion of symptomatic cases to the Omicron outbreak in Shanghai in March 2022 and estimated that the time-varying reproduction number = 3.4. National vaccination coverage as of March 2022 was used. The age-specific IFR and infection-hospitalization risk among unvaccinated individuals were derived from the Omicron wave in Hong Kong. |
| Leung, Leung, Wu [28] | Age-structured meta-population compartmental model | Effectiveness of 4^th^ dose in reducing hospitalizations and deaths, equivalent to 3 doses of inactivated vaccine against Omicron variant in Hong Kong [30]. Effectiveness of 3^rd^ dose against infection from a clinical trial in Pakistan and Malaysia [57]. Effectiveness of nirmatrelvir/ritonavir in reducing hospitalizations and deaths similar to that observed in Hong Kong. The effectiveness of NPI in reducing transmission potential is similar to that in Hong Kong. Used hospital capacity designated for COVID-19 from Hong Kong (100% & 200%) as a baseline and surged capacity benchmark in mainland China. Vaccine uptake rate in mainland China as of 28 Nov 2022. IFR data from England in 2020 and 2021 [58; 59]. They assumed that the hazard ratio of the Delta variant was 1.45 times that of the Alpha variant, and the hazard ratio of the Omicron variant was 0.3 times that of the Delta variant. |

IFR: Infection fatality ratio. NPI: Nonpharmaceutical interventions.

**Supplementary Table S14.** Summary of estimated death count (baseline scenario) and selected sensitivity/scenario analysis of two papers reviewed in Supplementary Text S3.

|  | Estimated death count (Baseline scenario) | Selected sensitivity/scenario analysis |
| --- | --- | --- |
| Cai et al. [26] | Over a 6-month period, 1.55 million deaths (1.10 per 1000) in the baseline scenario. | Simulations specific to 3 administrative regions (Shanghai, Shanxi, and Shandong), different scenarios of interventions (including different vaccination scenarios and NPI interventions), different effective reproduction numbers, and different combinations of these scenarios. |
| Leung, Leung, Wu [28] | 568 to 770 per million in Scenario 1 (status quo, with no 4^th^ dose vaccine, no antivirals, and no NPI). If this rate was applied to the age structure used in Cai et al., this would translate into a cumulative death count of 0.80 million to 1.09 million. | 5 scenarios. Scenario 1: Status quo; Scenario 2 included mass vaccination of the 4^th^ dose vaccine and a 60% coverage of antivirals. Scenario 3 included Scenario 2 and added NPI. Scenario 4 included Scenario 3 and with an improved vaccination rate. Scenario 5 included Scenario 4, but the 4^th^ dose could be any one of 5 (instead of 2) brands of vaccine (equal probability). |

NPI: Nonpharmaceutical interventions.

**Supplementary Table S15.** Age-stratified population size and coverage of inactivated COVID-19 vaccine primary series and booster in Cai et al. [26].

| Age group (years) | Population (2020) | Primary vaccine coverage (%) | Booster coverage (%) |
| --- | --- | --- | --- |
| 0-2 | 46,730,333 | 0.0 | 0.0 |
| 3-11 | 155,500,009 | 88.0 | 0.0 |
| 12-17 | 94,764,080 | 100.0 | 0.0 |
| 18-59 | 848,766,084 | 93.8 | 51.5 |
| 60-69 | 147,388,498 | 86.6 | 56.4 |
| ≥70 | 116,629,720 | 72.2 | 39.6 |
| Overall | 1,409,778,724 | 87.9 | 46.0 |

**Supplementary Table S16.** Parameter values applied in Cai et al. that determined the infection-fatality ratio (IFR) of the unvaccinated (data taken from Supplementary Table 8 of Cai et al. [26]). The terminology used is ours, instead of the original terms used in Cai et al. [26]. The IFR shown below is calculated for this systematic review using the equation:

$$IFR=\text{a×b×[c×(1-e)+d×e]}$$

Where a = infection-symptomatic ratio (unvaccinated), b=symptomatic-infection-hospitalization ratio (unvaccinated), c=Hospitalized-non-ICU-fatality ratio (unvaccinated), d=ICU-fatality ratio (unvaccinated), and e=Hospitalized-ICU ratio (unvaccinated)

| Age group (years) | Infection-symptomatic ratio (unvac.) (%) | Symptomatic-infection-hospitalization ratio (unvac.) (%) | Hospitalized-non-ICU-fatality ratio (unvac.) (%) | ICU-fatality ratio (unvac.) (%) | Hospitalized-ICU ratio (unvac.) (%) | IFR (unvac.) (%) |
| --- | --- | --- | --- | --- | --- | --- |
| 0-2 | 7.16 | 11.09 | 0.26 | 3.40 | 0.23 | 0.0021219 |
| 3-11 | ‘’ | 6.37 | 0.52 | 6.74 | 1.61 | 0.0028284 |
| 12-17 | ‘’ | 15.01 | 0.75 | 9.81 | 3.33 | 0.0113028 |
| 18-29 | 8.87 | 7.41 | 0.96 | 12.48 | 5.39 | 0.0103909 |
| 30-39 | ‘’ | 9.78 | 0.80 | 10.45 | 9.82 | 0.0151604 |
| 40-49 | 12.09 | 8.02 | 3.23 | 20.66 | 14.66 | 0.0560947 |
| 50-59 | ‘’ | 17.06 | 5.75 | 33.32 | 19.49 | 0.2294260 |
| 60-69 | 15.89 | 20.45 | 8.68 | 36.45 | 29.67 | 0.5497954 |
| ≥70 | ‘’ | 65.60 | 24.98 | 37.47 | 48.22 | 3.2316696 |

‘’: same as above. ICU: intensive care unit. IFR: infection-fatality ratio; unvac.: unvaccinated

**Supplementary Table S17.** The infection-hospitalization ratio and infection-fatality ratio (IFR) of unvaccinated individuals of a variant of concern similar to the Omicron variant, as applied in Leung et al. [28]. Leung et al. assumed that these numbers are Beta-distributed with a coefficient of variation of 0.05. They assumed that the hazard ratio of the Delta variant was 1.45 times that of the Alpha variant, and the hazard ratio of the Omicron variant was 0.3 times that of the Delta variant [28]. Leung et al.’s data sources are: [58; 59].

| Age group (years) | Infection-hospitalization ratio (unvaccinated) (%) | IFR (unvaccinated) (%) |
| --- | --- | --- |
| 0-9 | 0.011 | 0.0005 |
| 10-19 | 0.027 | 0.0005 |
| 20-29 | 0.72 | 0.0005 |
| 30-39 | 2.34 | 0.023 |
| 40-49 | 2.94 | 0.023 |
| 50-59 | 5.52 | 0.126 |
| 60-69 | 7.98 | 0.126 |
| 70-79 | 11.28 | 2.00 |
| ≥80 | 12.48 | 8.70 |

**Supplementary Table S18.** Population age structure used in Leung et al. [28]. They used the age structure data from China’s 2010 population census data [35].

| Age group (years) | Population size (2010) |
| --- | --- |
| 0-9 | 146,414,159 |
| 10-19 | 174,797,576 |
| 20-29 | 228,426,370 |
| 30-39 | 215,164,162 |
| 40-49 | 230,348,517 |
| 50-59 | 160,065,645 |
| 60-69 | 99,780,564 |
| 70-79 | 56,824,530 |
| ≥80 | 20,989,346 |
| TOTAL | 1,332,810,869 |
